# E3 Ubiquitin Ligases in Neurological Diseases: Focus on Gigaxonin and Autophagy

**DOI:** 10.3389/fphys.2020.01022

**Published:** 2020-10-22

**Authors:** Léa Lescouzères, Pascale Bomont

**Affiliations:** ATIP-Avenir Team, INM, INSERM, University of Montpellier, Montpellier, France

**Keywords:** Gigaxonin, E3 ligase, ubiquitin, neurodevelopmental disease, neurodegenerative disease, cytoskeleton, cell signaling, autophagy

## Abstract

Ubiquitination is a dynamic post-translational modification that regulates the fate of proteins and therefore modulates a myriad of cellular functions. At the last step of this sophisticated enzymatic cascade, E3 ubiquitin ligases selectively direct ubiquitin attachment to specific substrates. Altogether, the ∼800 distinct E3 ligases, combined to the exquisite variety of ubiquitin chains and types that can be formed at multiple sites on thousands of different substrates confer to ubiquitination versatility and infinite possibilities to control biological functions. E3 ubiquitin ligases have been shown to regulate behaviors of proteins, from their activation, trafficking, subcellular distribution, interaction with other proteins, to their final degradation. Largely known for tagging proteins for their degradation by the proteasome, E3 ligases also direct ubiquitinated proteins and more largely cellular content (organelles, ribosomes, etc.) to destruction by autophagy. This multi-step machinery involves the creation of double membrane autophagosomes in which engulfed material is degraded after fusion with lysosomes. Cooperating in sustaining homeostasis, actors of ubiquitination, proteasome and autophagy pathways are impaired or mutated in wide range of human diseases. From initial discovery of pathogenic mutations in the E3 ligase encoding for E6-AP in Angelman syndrome and Parkin in juvenile forms of Parkinson disease, the number of E3 ligases identified as causal gene for neurological diseases has considerably increased within the last years. In this review, we provide an overview of these diseases, by classifying the E3 ubiquitin ligase types and categorizing the neurological signs. We focus on the Gigaxonin-E3 ligase, mutated in giant axonal neuropathy and present a comprehensive analysis of the spectrum of mutations and the recent biological models that permitted to uncover novel mechanisms of action. Then, we discuss the common functions shared by Gigaxonin and the other E3 ligases in cytoskeleton architecture, cell signaling and autophagy. In particular, we emphasize their pivotal roles in controlling multiple steps of the autophagy pathway. In light of the various targets and extending functions sustained by a single E3 ligase, we finally discuss the challenge in understanding the complex pathological cascade underlying disease and in designing therapeutic approaches that can apprehend this complexity.

## Introduction: E3 Ubiquitin Ligases Identified as Causal Gene Products in Neurological Diseases

**FIGURE 1:**
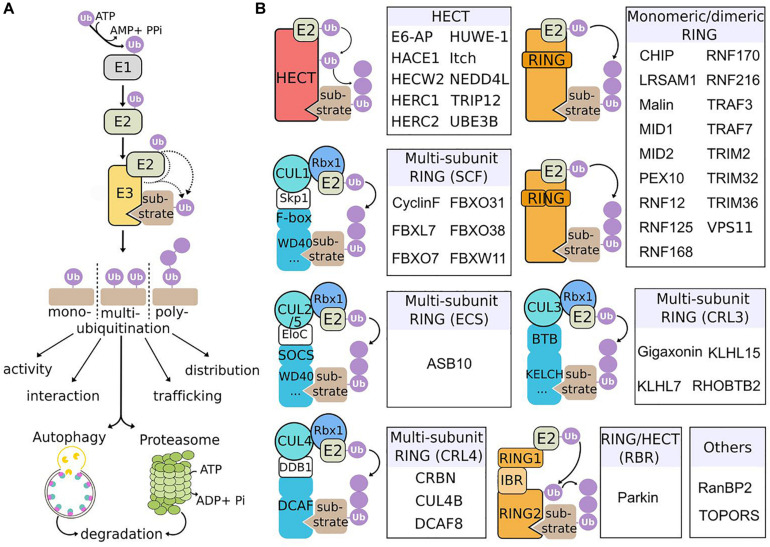
Mechanism of action and classification of E3 ubiquitin ligases causing neurological diseases in human. **(A)** Ubiquitination cascade. E1 enzymes activate ubiquitin (Ub) in an ATP-dependent manner, forming a thioester bound with Ub. Activated-Ub is then transferred to Ub-conjugating enzymes (E2). Finally, E3 ubiquitin ligases assist or directly catalyze the transfer of Ub to substrates. Substrates can be mono-, multi- or poly-ubiquitinated, with different types of Ub chains, to modulate various processes, including protein activation, interaction, trafficking, subcellular localization, and degradation through the 26S proteasome or the autophagy pathway. **(B)** E3 ligases underlying neurological diseases and their classification. The three classes of E3 (HECT, RING, and RING-HECT hybrid) are represented. E3 ligases identified as the genetic cause of neurodevelopmental and neurodegenerative diseases in human are classified within each category (sub-classes with no disease representative are not shown). The HECT and RING E3 ligases differ in the way they transfer Ub to the substrate. The HECT E3s receive Ub from E2 enzymes, form a thioester intermediate with Ub, and catalyze the transfer to the substrate. RING E3s associate with E2 enzymes and substrates and mediate the transfer of Ub from E2 to the substrate. RING E3s can function as monomers, dimers or multi-subunit RING E3s. The latter class is subdivided in different groups, among which the Cullin-RING Ligases (CRLs) are the largest E3 ligase family. SCF, ECS, CRL3 and CRL4 ligases are composed of a modular E3 core containing specific Cullins (respectively CUL1, CUL2/5, CUL3, CUL4) and RBX1 (RING-box1 proteins), and a substrate specificity module composed of an adaptor (Skp1, EloC, and DDB1) and a substrate receptor. The RING-HECT hybrid ligases (RBR for RING-between-RING) interact with E2 as the RING E3 but transfer Ub with HECT-like mechanism. Finally, particular E3 are represented (“Others”): RanBP2 does not transfer Ub but Sumo (small ubiquitin-related modifier) and is neither an HECT, nor a RING E3 ligase; TOPORS has a RING domain and is a dual E3 Ub and Sumo ligase.

E3 ubiquitin ligases constitute a large family of enzymes that play pivotal roles in protein ubiquitination, a major posttranslational modification regulating various cellular functions, as diverse as DNA repair, proliferation, apoptosis, transcription, circadian clock, endocytosis, cell signaling, immunity, and protein quality control (Swatek and Komander, 2016). Ubiquitination involves a cascade of enzymatic reactions, driven by ubiquitin-activating enzymes (E1), ubiquitin-conjugating enzymes (E2) and ubiquitin-ligases (E3) that ultimately transfer the ubiquitin moieties to specific targets (Komander and Rape, 2012; Figure 1A). Represented by ∼800 distinct genes, E3 ligases provide an exquisite precise and diverse mode of control of cellular processes, through the spatial, temporal and substrate specificity of the E3 ligases, and the variety of ubiquitination types (mono, multi, and poly) and ubiquitin chains (on the 7 Lys residues or the N-terminal Met of ubiquitin) (Kwon and Ciechanover, 2017). This diversity underlies the multiples roles of ubiquitination in regulating the fate of proteins, from their activity, interaction, trafficking, subcellular distribution, and degradation. Ubiquitinated substrates can be degraded by the proteasome and/or autophagy, two pathways that cooperate to maintain cellular and tissue homeostasis but that bear distinct properties (Kocaturk and Gozuacik, 2018). Proteasome is a multi-catalytic protease complex essential to degrade short-lived proteins or misfolded/damaged proteins, whereas autophagy preferentially eliminates long-lived proteins, insoluble protein aggregates and also organelles and parasites (Rousseau and Bertolotti, 2018). The (macro)autophagy machinery is a multistep process leading to the engulfment of material in a double membrane vesicle called autophagosomes, that subsequently fuse to lysosomes for degradation of content by lysosomal enzyme (Klionsky and Emr, 2000; Galluzzi et al., 2017). While degradation can be independent of ubiquitination (called bulk autophagy), ubiquitin signaling triggers a specific response (called selective autophagy), to clear identified material. Cooperating with proteasome to clear damaged or old cellular compounds in basal conditions, autophagy is upregulated upon stress (nutritional, hypoxia, and chemical) to recycle material and therefore provide nutrients essential for adaptation and survival. Thus, the ubiquitin proteasome system (UPS) and autophagy are crucial for physiology and alterations of these machineries underlie a wide range of human diseases, including cancer, immune and neurodegenerative diseases (Menzies et al., 2017; Rousseau and Bertolotti, 2018; Levine and Kroemer, 2019).

**FIGURE 2:**
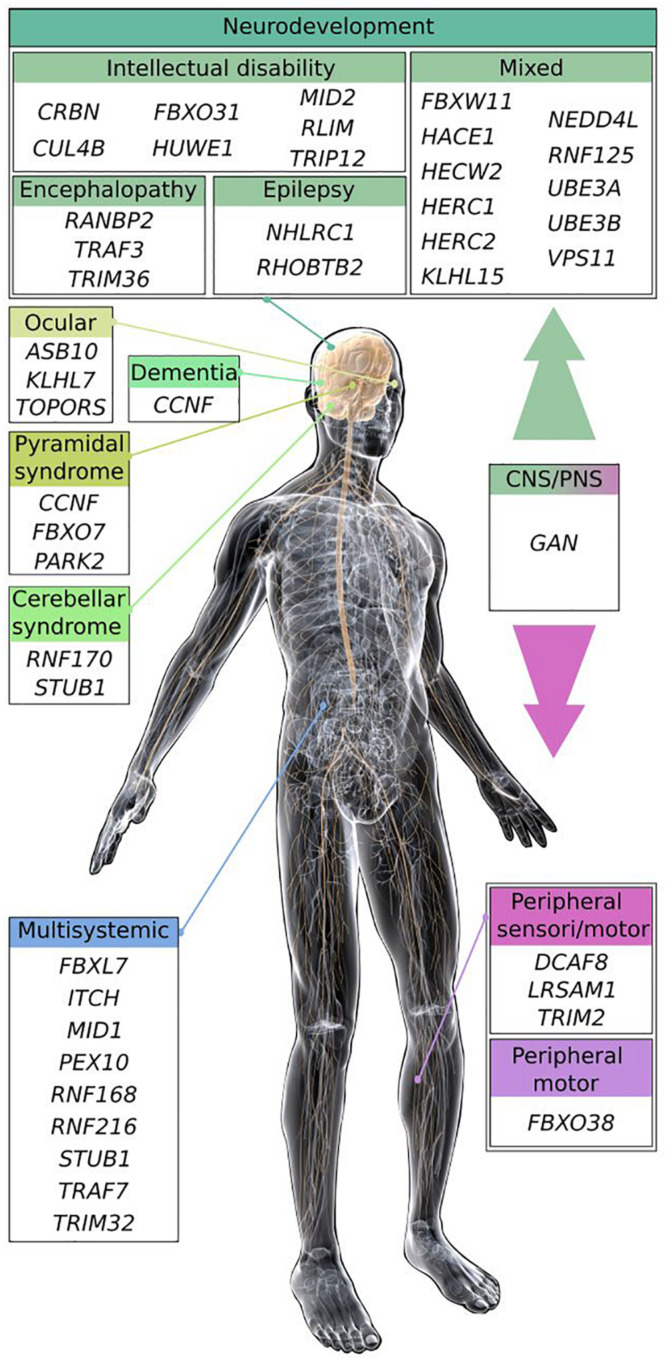
Scheme of the main clinical features shared by E3 ligase encoding genes causing neurodevelopmental and neurodegenerative diseases. Most of the E3 ligase genes, when mutated cause neurodevelopmental diseases (intellectual disability, encephalopathy, and epilepsy), or regionalized neuropathies (cerebellar, ocular, and pyramidal syndrome) within the CNS. Some pathologies exhibit mixed symptoms that can be part of broader multisystemic disorders. E3 ligase genes inducing peripheral neuropathies are represented by Charcot-Marie-Tooth diseases (CMT), and distal hereditary motor neuropathy (HMN). The GAN E3 ligase gene causes a disease affecting broadly the nervous system, leading to a myriad of symptoms within the peripheral and the CNS. Illustration: © www.gograph.com/ Eraxion. Genes (protein if different), names of diseases and references (in alphabetical order): *ASB10*, Glaucoma (Pasutto et al., 2012); *CCNF* (cyclinF), Amyotrophic Lateral Sclerosis and Frontotemporal dementia (Williams et al., 2016); *CRBN*, mental retardation (MRT2A; Higgins et al., 2004); *CUL4B*, mental retardation (MRXS15; Zou et al., 2007); *DCAF8*, Charcot-Marie-Tooth disease (CMT2; Klein et al., 2014); *FBXL7*, Hennekam syndrome (Boone et al., 2020); *FBXO7*, Parkinson syndrome (PARK15; Shojaee et al., 2008); *FBXO31*, mental retardation (MRT45; Mir et al., 2014); *FBXO38*, distal hereditary motor neuropathy (HMN2D; Sumner et al., 2013); *FBXW11*, neurodevelopmental syndrome (Holt et al., 2019); *GAN* (Gigaxonin), Giant Axonal Neuropathy (Bomont et al., 2000); *HACE1*, neurodevelopmental syndrome (SPPRS; Hollstein et al., 2015); *HECW2*, neurodevelopmental syndrome (NDHSAL; Berko et al., 2017); *HERC1*, neurodevelopmental syndrome (MDFPMR; Nguyen et al., 2016); *HERC2*, mixed with mental retardation (MRT38; Puffenberger et al., 2012); *HUWE1*, mental retardation (MRXST; Froyen et al., 2008); *ITCH* (Itch), multi-system autoimmune disease with neurodevelopmental defects (Lohr et al., 2010); *KLHL7*, retinitis pigmentosa (RP42; Friedman et al., 2009); *KLHL15*, mixed with mental retardation (MRX103; Mignon-Ravix et al., 2014); *LRSAM1*, CMT2P (Guernsey et al., 2010); *MID1*, Opitz G/BBB syndrome 1 (GBBB1; Quaderi et al., 1997); *MID2*, mental retardation (MRX101; Geetha et al., 2014); *NEDD4L*, Periventricular nodular heterotopia 7 (PVNH7; Broix et al., 2016); *NHLRC1* (Malin), Lafora disease (Chan et al., 2003); *PARK2* (Parkin), Parkinson disease 2 (PARK2; Kitada et al., 1998); *PEX10*, Zellweger syndrome (Okumoto et al., 1998; Warren et al., 1998); RANBP2* (RanBP2), acute necrotizing encephalopathy (Neilson et al., 2009); *RHOBTB2*, epileptic encephalopathy (EIEE64; Belal et al., 2018); *RLIM* (RNF12) intellectual disability (Tonne et al., 2015); *RNF125*, overgrowth syndrome (Tenorio et al., 2014); *RNF168*, RIDDLE syndrome (Stewart et al., 2009); *RNF170*, sensory ataxia (SNAX1; Valdmanis et al., 2011); *RNF216*, Gordon Holmes syndrome (GHS; Margolin et al., 2013); *STUB1* (CHIP), Spinocerebellar ataxia (SCAR16, SCA48, and GHS; Shi et al., 2013, 2014; Genis et al., 2018); *TOPORS*, retinitis pigmentosa (RP31; Chakarova et al., 2007); TRAF3*, encephalopathy (Perez de Diego et al., 2010); *TRAF7*, multisystem disorder with neurodevelopmental delay (Tokita et al., 2018); *TRIM2*, CMT2R (Ylikallio et al., 2013); *TRIM32*, Bardet-Biedl syndrome (BBS11; Chiang et al., 2006); *TRIM36*, anencephaly (Singh et al., 2017); *TRIP12*, intellectual disability with or without autism (O’Roak et al., 2014; Lelieveld et al., 2016); *UBE3A* (E6-AP), Angelman syndrome (Kishino et al., 1997; Matsuura et al., 1997); *UBE3B*, Kaufman oculocerebrofacial syndrome (KOS; Flex et al., 2013); *VPS11*, neurodevelopmental and leukoencephalopathy (Edvardson et al., 2015; Zhang et al., 2016). Asterisk (*) are susceptibility genes for viral-induced neuropathies.

Conferring substrate specificity for ubiquitination, E3 ligases are key in regulating protein activation, function, and degradation. There are classified into three major groups: the HECT (homologous to E6-AP carboxyl terminus), the RING (really interesting new gene) and the RING-HECT hybrid E3s (Zheng and Shabek, 2017; Figure 1B). The HECT domain E3 ligases catalyze the attachment of ubiquitin to the substrate, while the RING finger E3 ligases do not have a catalytic role and act as scaffold to bridge E2 and substrate for the transfer of ubiquitin from the E2 to the substrate. RING E3s constitute a large family, which is formed of monomeric RINGs, dimeric RINGs or multi-subunit RINGs assembled around Cullin subunits. Hybrid E3s, the RING-HECT ligases interact with E2 as the RING enzymes but transfer ubiquitin with HECT-like mechanism. The last years have seen a rise in the identification of E3 ligase encoding genes as the cause of neurological diseases in human. Since the identification of the first HECT (E6-AP in Angelman syndrome; AS) and RING (Parkin in juvenile form of Parkinson disease) E3 ligases (Kishino et al., 1997; Matsuura et al., 1997; Kitada et al., 1998), 42 additional players are shaping the diversified landscape of E3 types in the origin of neurological diseases (Figure 1B). These diseases, mostly inherited by a recessive mode for a general loss-of-function mechanism of the respective E3 ligases are extremely diverse. The spectrum spreads from neurodevelopmental to adult neurodegenerative pathologies, including various forms of intellectual disability, encephalopathy, epilepsy, retinitis pigmentosa, ataxia, and Parkinson’s disease (Figure 2). These clinical features can be mixed in various neurodevelopmental conditions like AS, or represent one component of broader multisystemic diseases, like Opitz G/BBB and Bardet–Biedl syndromes. E3 ligases mutated in peripheral neuropathies are exemplified by several forms of Charcot-Marie-Tooth (CMT) diseases and distal hereditary motor neuropathy. While rare cases of peripheral neuropathies exhibit some central signs [TRIM2 with vocal cord paralysis (Pehlivan et al., 2015) and LRSAM1 with Parkinson features (Aerts et al., 2016)], Giant Axonal Neuropathy (GAN), caused by a CRL3 adaptor named Gigaxonin stands for the spreading of symptoms across neuronal tissues in both peripheral nervous system (PNS) and central nervous system (CNS). In this review, we present the genetic underlying GAN, the generation of novel biological models and the recent advances into the key functions of Gigaxonin. With this focus on Gigaxonin, we then provide a comprehensive analysis of common functional themes shared with other E3 ligases in controlling pivotal cellular pathways: cytoskeleton organization, cell signaling, and autophagy. Expanding on the later, we discuss how E3 ligases mutated in neurological diseases directly modulate multiple steps of the autophagy pathway, hence providing novel opportunities for the identification of specific spots for therapeutic intervention in neurological diseases, and more largely for the use of these E3 ligases in developing novel tools for the benefit of many other diseases.

## Gigaxonin Encoding Gene, Genetic Cause of Giant Axonal Neuropathy

### Giant Axonal Neuropathy

**FIGURE 3:**
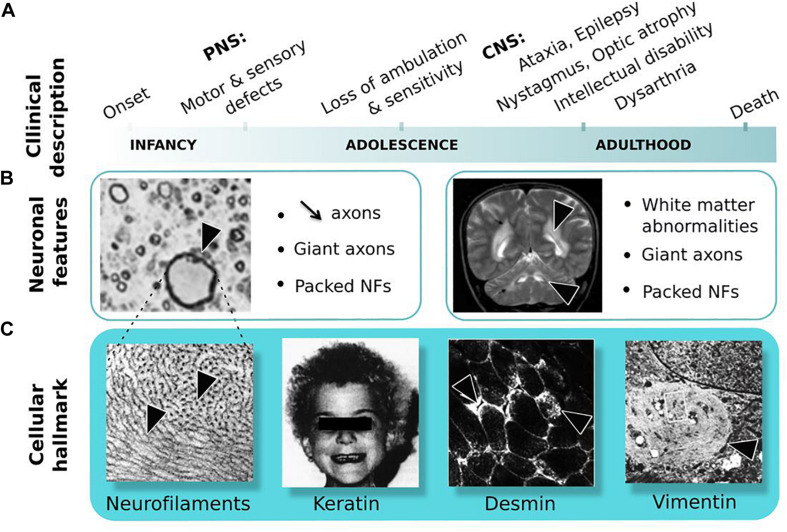
Giant Axonal Neuropathy. **(A)** Symptoms are shown in order of occurrence: from onset in childhood in the peripheral nervous system (PNS) to adulthood in the central nervous system (CNS). **(B)** Neuronal features: (left) nerve cross-section in the PNS shows reduced axonal content and giant axon; (right) magnetic resonance imaging of the brain reveals white matter abnormalities. Giant axons of CNS are not represented here. **(C)** Cellular hallmark: (from left to right) accumulation and altered distribution of neurofilaments (NFs) in giant axons, curled hair, desmin bundles in muscles and aggregates of vimentin in skin-derived fibroblasts. Copyright with permission from BioMed Central, Springer and Elsevier.

Giant axonal neuropathy (GAN, MIM#256850) is a rare neurodegenerative disease with an autosomic and recessive mode of inheritance (Asbury et al., 1972; Berg et al., 1972). This review will not provide an exhaustive presentation of clinical symptoms, which has been described elsewhere (Kuhlenbaumer et al., 1993; Johnson-Kerner et al., 2014) but will highlight key features. Giant axonal neuropathy is unique, for its wide alteration of the nervous system and its severity (Figure 3A). In the classical severe form, GAN starts in infancy, touches both sensory and motor modalities of the PNS, evolves toward a loss complete of ambulation and sensitivity during adolescence, and subsequently spreads to the CNS during adulthood. The outcome is fatale in young adult, usually before the third decade. Few milder cases have been described, varying in disease onset and presenting slow progression with, in certain cases no overt alteration of the CNS (see Supplementary Table 1). Underlying this massive deterioration of nervous functions are the decreased axonal density and presence of enlarged “giant” axons throughout the nervous system (Figure 3B). Giant axons are filled with abnormally packed neurofilaments, the neuronal Intermediate Filaments (IFs) that constitute the most abundant cytoskeletal component of the nervous system. Notably, IF aggregation extends beyond the nervous system, touching other IF types as keratin, desmin, and vimentin (Figure 3C), hence placing GAN as a disease of the cytoskeletal IFs. Extremely severe, GAN still shares at early stage features with several forms of CMT diseases, that present similar sensory-motor deficits, giant axons, and NF aggregation (Fabrizi et al., 2004; Azzedine et al., 2006; Ylikallio et al., 2013; Klein et al., 2014).

### The *GAN* Gene: Transmission and Mutations

**FIGURE 4:**
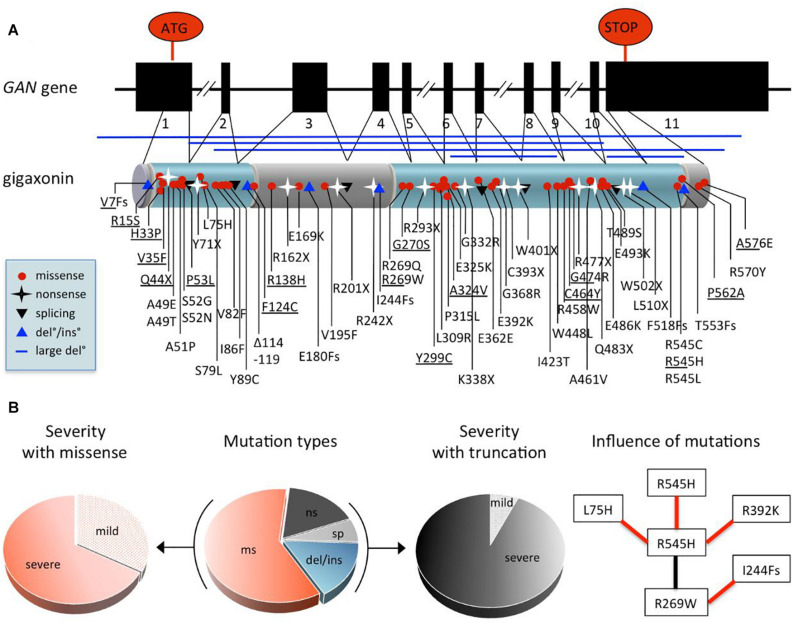
Comprehensive analysis of *GAN* mutations. **(A)** Exon-intron structure of the *GAN* gene and domain organization of the encoded protein Gigaxonin, with a N-terminal BTB domain and a C-terminal Kelch domain (depicted in blue, linker is called BACK domain). Mapping of the 75 mutations (see also Supplementary Table 1 for details): missense mutation (red circle), nonsense mutations (black star), mutations at splicing sites (black triangle), deletions/insertion (blue triangle) and large deletion (blue line). **(B)** Pie charts depicting the effects of GAN mutations. Distribution of mutation types in patients (middle): 60% are missense mutations “ms,” 17.4% of nonsense mutations “ns,” 16% of deletions/insertions “del/ins,” and 6.6% mutations at donor/accepting splicing sites “sp.” Other pie charts indicate the predominance of severe forms associated with missense mutations (left) and truncations [(right), including “ns,” “sp,” “del/ins”], representing 67.7 and 93.3% of cases, respectively. On (right), flow chart showing the influence of mutations on severity. Black and red lines indicate, respectively that the patient bearing both mutations has a mild or severe form of the disease. As example, the R545H mutation leads to a severe phenotype when found homozygous, while it is attenuated by combination with R269W, which is severe when associated to I244Fs.

While gene identification can be achieved through direct sequencing of the human genome nowadays, the *GAN* gene was discovery through a two steps process, comprising genetic mapping and screening of potential coding sequences within the interval co-segregating with the disease. As for many rare recessively inherited-disease, the genetic localization of the *GAN* gene was made possible thanks to homozygosity mapping, in consanguineous families that present a higher risk of transmitting mutated alleles. Thus, the GAN locus was delineated within the cytogenetic portion of chromosome 16 in 16q24.1 (Ben Hamida et al., 1997; Flanigan et al., 1998) with borders being further reduced thanks to the concomitant identification of novel polymorphic markers and other families (Cavalier et al., 2000). Subsequently, a bioinformatic methodology was developed to make profit of the partial and fragmented genomic sequences released by the ongoing Human Genome sequencing initiative. This pioneer in silico approach (as acknowledged in Lander et al., 2001) led to the discovery of the *GAN* gene subsequently to identification, extension of novel coding portions within the GAN interval and screening for mutations in patients (Bomont et al., 2000). With the first genetic variant identified in the short ESTaa306952, the full 1791 bp coding sequence was cloned and shown to encompass 11 exons. The novel protein, named Gigaxonin was shown to contain a BTB domain at its N-terminus and a Kelch domain, composed of 6 Kelch repetitions at its C-terminal end (Bomont et al., 2000; Figure 4A).

In the initial study, a total of 14 distinct pathogenic variants were identified in 12 families, including 2 homozygous truncating mutations, hence unambiguously defining Gigaxonin as the GAN causing gene product. This original publication, which highlighted a large spectrum of mutation types and a scattering along the entire gene has been expanded by various laboratories worldwide. Today, 75 distinct mutations have been identified in 75 families from various geographic origins, at homozygous and heterozygous state (Supplementary Table 1). The major genetic alterations are missense mutations (60%), the remaining causing truncation of Gigaxonin, due to nonsense mutations (17.4%), deletions/insertions (from small amino acids to the entire gene) (16%), and mutations at donor/accepting splicing sites (6.6%).

While most mutations are unique (81.3%), others are enriched in given populations (R293X and IVS9+1G > T in Turkey, R477X in Algeria), probably as a result of the spreading of common alleles through consanguinity. Several mutations are shared by different countries, that could indicate an ancestral funder effect but could also represent independent mutational “spots”: S79L, R162X, R242X, R269W, P315L, G368R, G474R, E486K, E493K, R545H, R477X, A576E, as well as V7Fs-ins/del, A49E/T, S52G/N, and R545C/H/L. Altogether, these genetic studies have revealed that no major hot spot is found in GAN and that no exon is left intact. As a result, genetic diagnosis for GAN requires the full sequencing of the gene, complemented by cytogenetic analysis for large deletions. In regard to the various phenotypic expression of GAN, which is extremely severe in the vast majority of cases but can exhibit milder forms, assumption is often made to correlate mutation type and position with severity. This is very hazardous, and certainly neither missense, nor position outside the BTB or Kelch domains is synonymous of mild. They are many examples and counter-examples in GAN to show that this is not that simple. Moreover, inter and intra-familial clinical heterogeneity has been evidenced for patients bearing the same mutation (Tazir et al., 2009), hence suggesting that genetic alteration might not be the only determinant of clinical expression. While speculation on the effect of individual mutation is difficult to make, the global analysis of data can permit to point out general features (Figure 4B). First, the majority (67%) of missense mutations are associated to severe forms of GAN, the remaining contributing to 88% of the mild forms. Indeed, except for two truncating mutations (compound heterozygous V7Fs/Y299C and Q44X/G474R), all mutations associated with mild forms are composed of missense mutations (homozygous or heterozygous). Second, with the exceptions of V7Fs and Q44X cited above, all truncating mutations are associated with severe forms of GAN. Third, the analysis of shared mutations between families of various origins is very informative. Indeed, in four cases, families sharing one common mutation (R269W, G474R, R545H, or A576E) in association with distinct ones show different severity (see example in Figure 4B). Thus, with the caution that mutation may not be the only determinant of severity, this might tell us that mutation can affect one another to modulate Gigaxonin functionality, at the protein and possibly mRNA level. Another characteristic of GAN was suggested as a potential predictive marker of the mild cases: the hair. Indeed, while all GAN patients present giant axons filled with abnormally bundled NFs at the nerve biopsy, few mild cases were associated to normal hair among patients with kinky hairs (Figure 3C and Supplementary Table 1). Here, the analysis of both criteria reveals that “normal hair” is equally found in mildly or severely affected patients but that most of mild cases have normal hair. This implies that the criteria “normal hair” can’t be used as a predictive marker of disease severity, at least in correlation with general clinical presentation that can vary between laboratories.

## Gigaxonin, Substrate Adaptor of Cul3-E3 Ligase

### Novel BTB-Kelch Protein and Structure

**FIGURE 5:**
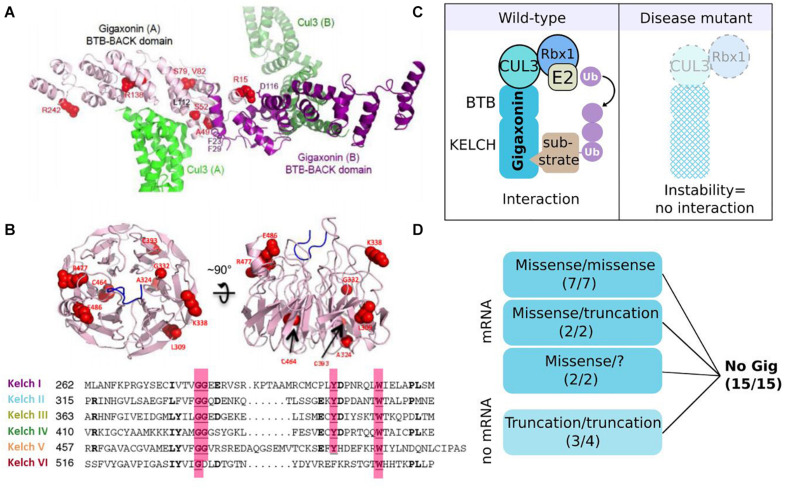
Gigaxonin-E3 ligase: protein structure and instability as a general effect of mutations. **(A)** Structural model of the BTB-BACK homodimer of Gigaxonin (purple), in complex with Cul3 (green). Some mutations are represented in red (Boizot et al., 2014). **(B)** Representation of the top and side views of a structural model for the β-propeller Kelch domain of Gigaxonin, whose 6-blades are shown in sequence alignment below. Some mutations are represented in red. **(C)** In wild-type condition (left) Gigaxonin interacts with Cul3 through its N-terminal BTB domain and binds to substrates through its C-terminal Kelch domain, hence catalyzing the transfer of ubiquitin chain from the E2 to the recognized substrate. In disease (right), *GAN* mutations induce a general instability of Gigaxonin, by affecting the proper folding of the BTB or Kelch domain, or impairing homodimerization and interaction with Cul3. **(D)** Dramatic reduction of Gigaxonin levels in patient’s cells: due to instability of the mutated protein, or degradation of the corresponding mRNA, depending on the mutation type and combination. “?”: compound heterozygous mutations await to be discovered. Copyright with permission from BioMed Central.

Gigaxonin is a 597 amino acid long BTB-Kelch protein (GenBank ID: mRNA AF291673, protein protein_id = AAG35311) belonging to the Cullin 3-RING (CRL3) subgroup of E3 ubiquitin ligases (Figures 1, 5C). This link is mediated by the BTB domain, and was established by the key findings of the (i) structural similarity of the BTB domain-fold with Skp1/elonginC (Schulman et al., 2000) and (ii) specific interaction of BTB containing proteins with Cul3 (Furukawa et al., 2003; Pintard et al., 2003; Xu et al., 2003). This central work demonstrated that BTB family members are the substrate-specific adaptors of Cul3 E3 ligases, and that Gigaxonin indeed interacts with Cul3 (Furukawa et al., 2003). As known for transcription factors bearing a BTB domain, Gigaxonin BTB domain was shown to homodimerize (Cullen et al., 2004), a process that is predicted to be of functional importance for E3 ligase activity. Thus, the C-terminal Kelch domain of Gigaxonin, which is composed of 6 Kelch motifs, would serve as a binding domain for substrates. The x-ray crystal structure of another BTB-Kelch protein (Keap1) revealed that the Kelch domain forms a ß-propeller structure in which individual repeats constitute the blades (Li X. et al., 2004). This tri-dimensional structure generates multiple surfaces for protein–protein interaction. The structure of Gigaxonin was solved by crystallography for its BTB portion (Zhuang et al., 2009) and modelized for its interface with Cul3 and its Kelch domain (Boizot et al., 2014) (Figures 5A,B). This model represents the dimers of Gigaxonin in interaction with Cul3 and the ß-propeller structure formed by the six Kelch repetitions, exposing interacting surfaces at the top, the bottom, and the circumference. In this three-dimensional organization, the interaction motif for a given substrate may involve amino acids spatially close (intra Kelch) or very distant (inter Kelch). This, combined to the low identity between individual Kelch repeats hampers the prediction of putative partners for Kelch proteins by sequence alignment but surely generates a high diversity of interaction amongst Kelch proteins. So far, seven substrates have been identified for Gigaxonin and their identity and functions will be detailed in the “Gigaxonin functions” part.

### General Effects of Mutations on mRNA/Protein Stability

Giant axonal neuropathy is a recessive disease and so far, Gigaxonin abundance is found systematically reduced in patients (Figure 5D), as seen on immunoblots with the specific monoclonal antibodies Gig^A^ on various immortalized lymphoblast cells (Cleveland et al., 2009). This method has been challenged and validated as a powerful research tool for the differential diagnosis of GAN (Boizot et al., 2014), whose clinical and histopathological features are overlapping with several forms of CMT diseases. Pulse-chase labeling of Gigaxonin overexpressing mutants demonstrated that regardless of the mutation type and the associated severity of the pathology, Gigaxonin mutants exhibit a shorter half-life (1–3 h) in comparison to the wild-type protein (10 h), suggesting a general mechanism of protein instability. It is important to note that while overexpression studies are useful to characterize the protein and its interaction with substrates for example, it certainly can’t be used to conclude on the physiology, nor on the functional roles of specific mutants. Indeed, reduced Gigaxonin amount (as seen in patients) is not equal to a massive expression of a mutant, whose shorter half-life is masked by continuous expression. Moreover, pulse-chase experiments revealed that mutant levels exhibit a high variation at 0 h, hence pointing out that results on different mutant/wild-type proteins can’t be compared without adjusting their expression levels. To investigate the causes of the generalized instability of Gigaxonin mutants, 19 mutations from 15 families (with mild or severe GAN) were mapped onto the structural model. With two exceptions for which hypothesis was challenging, this analysis predicted a general instability of the three-dimensional structure of the mutated proteins. Mainly, mutations would affect the proper folding of the BTB or Kelch domain, and could also impair interaction with the BTB domain or Cul3, all conditions expected to induce the degradation of mutated Gigaxonin. Additional examination of mRNA levels from patient cells revealed that truncating mutations have barely detectable mRNA (Figure 5D), probably as a result of a mechanism of mRNA quality control called non-sense mediated mRNA decay. Interestingly, when combined to a missense mutation (in two heterozygous compound mutants), truncating mutation present with normal amount of mRNA level, indicating that the missense mRNA may either be increasingly transcribed or exhibit a dominant positive effect on the truncated one. As discussed above in the genetic part, this also suggests that mutation can affect one another, here to modulate Gigaxonin level.

Altogether, our current knowledge points to a general effect of *GAN* mutations on instability, hence disrupting the global interactome of Gigaxonin in all patients (Figure 5C). This indicates that continuous overload of overexpressed mutants (which maintain a high level of protein despite the instability of the mutants) should not be used to demonstrate disease specificity, as mutants most probably conserve their interaction with partners.

### Gigaxonin Localization and Expression Pattern

Determining tissular, cellular and subcellular localization of a protein contributes tremendously to the investigation of its function(s). For Gigaxonin, 20 years of research have not quite answered the question. Over 50 monoclonal and polyclonal antibodies have been produced by several laboratories or from commercial sources. With the caution that should be made towards overexpression system, ectopic Gigaxonin produces a wide range of granular staining depending on the cell type (Bomont and Koenig, 2003), and its putative localization to the Golgi apparatus (Cullen et al., 2004) has not been reproduced (Bomont, 2016). For subcellular localization of the endogenous Gigaxonin, the outcome is that no one demonstrated specificity: not only the publications reporting subcellular localization of Gigaxonin in neuronal tissues (Ding et al., 2002; Cullen et al., 2004) did not provide internal controls but the comparison of immunostaining using many antibodies (including the ones cited above) with patient cells and tissues/cells from the GAN knock-out mice evidenced aspecific detection (Bomont, 2016). Incorrect statements on the localization of Gigaxonin are due to the detection of major aspecific band(s) by many (non)-commercial antibodies, as revealed by immunoblotting method (Bomont, 2016). The systematic analysis using samples from patients and 2 independent GAN mice shows that the endogenous Gigaxonin is a 65kDa predicted protein that migrates between 55 and 62 kDa (Dequen et al., 2008; Cleveland et al., 2009; Ganay et al., 2011; Boizot et al., 2014). To our knowledge, only the Gig^A^ monoclonal antibody detects endogenous Gigaxonin with a unique band (Cleveland et al., 2009), and still, this valuable reagent was not able to reveal the subcellular localization of Gigaxonin. This is most certainly attributed to the low level of expression of this E3 ligase adaptor, hence constituting an intracellular limiting factor to regulate the activity of this multi-subunit Cul3-E3 complex. Indeed, Gigaxonin was shown to account for 1.25 10^–3^% of total proteins in tissues (detergent soluble lysates of mouse brain) and 7500 molecules per cell (human lymphoblast cells) (Cleveland et al., 2009). Moreover, Gigaxonin was shown to be equally expressed in neuronal tissues, with higher degree in prenatal stages, but was also detected to a lower extend in non-neuronal adult tissues (Ganay et al., 2011).

## Biological Models To Study The GAN Pathology

**FIGURE 6:**
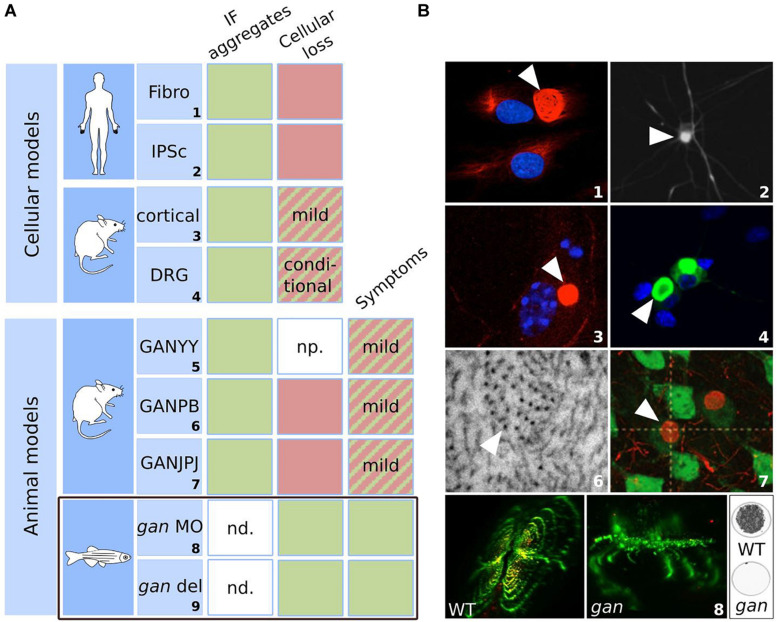
Biological models for GAN: strengths and weaknesses. Summary table **(A)** and illustrations **(B)** of the cellular and animal models developed for GAN. The table highlights their ability to reproduce the cellular marker and symptoms of the pathology. Green: the model satisfies the criteria; red: the model does not meet the criteria; hatched: the model partially satisfies the criteria. IF aggregation is evidenced in all cellular and animal models. Cellular loss was observed in aging GAN cortical neurons and upon specific conditions in DRG neurons. While GAN mice exhibit only modest symptoms, the *gan* zebrafish stands out as the first robust animal model for GAN, reproducing both neuronal loss and loss of motility, as in patients. np. not provided; nd: not determined. *gan* MO: transient repression using morpholino antisense oligonucleotide; *gan* del: genetic deletion mutant of *gan*. Numbers correspond to original publications: **1**: patient fibroblasts with bundling of vimentin (Pena, 1981; Bomont and Koenig, 2003); **2**: iPSC-derived motor neurons from GAN patients exhibiting peripherin aggregation (Johnson-Kerner et al., 2015a); **3**: GAN^–/–^ cortical neurons showing NF aggregate (Bomont: personal data). **4**: GAN DRG neurons with accumulations of α-internexin (Israeli et al., 2016); **5**: GAN1^Δ*ex*3–5^ (Ding et al., 2006); **6**: Electron micrograph of the axoplasm of GAN2^Δ*ex*3–5^ nerves (Ganay et al., 2011), showing abnormal packing and orientation of NFs; **7**: NF inclusions in GAN^Δ*ex*1^ brain neurons (Dequen et al., 2008); **8**: *gan* zebrafish exhibit abnormal spinal cord architecture, abolished neuromuscular junctions (left) and impaired locomotion (right: cumulative movement of larvae in a well during 1 h). Copyright with permission from Oxford University Press, BioMed Central (John Wiley & Sons) and J Clin Invest.

Several complementary biological models have been generated for GAN, to study the disease mechanisms but also to provide robust systems for therapeutic development. Thus, tracers of the pathology have been very useful to validate or invalidate these novel tools. As discussed earlier, the most manifest cellular hallmark of the GAN pathology is the wide bundling of the IF cytoskeletal network throughout tissues. At the physiological level, alterations of the sensory and motor systems, leading to loss of sensitivity and motility in patients during adolescence are the common peripheral pathological signs that have been used to evaluate the robustness of animal models. Here, we describe the different GAN systems, present their advantages and limitations (Figure 6) and how they were instrumental in providing crucial insights into Gigaxonin functions.

The first cellular model developed for GAN is the primary fibroblasts derived from skin biopsies of patients (Pena, 1981). Abnormal bundling of vimentin IF was evidenced in patient’s cells, while actin and microtubule networks seem unaltered. After the discovery of the *GAN* gene, analysis of several patients with identified mutations revealed that the ovoid perinuclear bundles, highly resistant to detergent is partial, conditional and dynamic (illustration no. 1 in Figure 6B) (Bomont and Koenig, 2003). First, in normal culture conditions, aggregates coexist with well-formed vimentin network and are detected in only 3–15% of total cells. Second, this proportion can considerably vary between laboratories for the same patient (Pena et al., 1983; Klymkowsky and Plummer, 1985), which seems to depend on culture conditions. The most striking demonstration was the quantitative and qualitative changes using low serum and confluency. Indeed, in these conditions (and independently of cell cycle stage), not only the proportion of aggregates increased by 5–20 fold, but aggregation forms a perinuclear ring cage that recruits all vimentin (Bomont and Koenig, 2003). This cytoskeletal alteration is specific for IF, as actin and microtubules are intact in GAN cells. In fact, microtubules have been shown to preserve cells from a total collapse of vimentin in GAN fibroblasts (Bomont and Koenig, 2003; Cleveland et al., 2009). Third, vimentin alteration in GAN cells is a dynamic process that can be exacerbated or reversed within day(s). While this cellular model is not valid to study neuronal alterations and exhibits a high interclonal variability, it represents the only naïve source from patient. The GAN fibroblast model was very crucial in identifying the role of Gigaxonin in controlling IF degradation (details on mechanistic in the next section).

The skin fibroblasts were also used to produce neurons, through differentiation of induced pluripotent stem cells (iPSCs) from patients. Thus, iPSC-derived motor neurons from distinct patients exhibit 3–4 fold increased in the abundance of the light-neurofilament protein (NFL), and more modestly peripherin, which still aggregates in motor neurons (illustration no. 2 in Figure 6B) (Johnson-Kerner et al., 2015a). The iPSCs are the only neuronal source derived from patients and were useful to confirm the aggregation of neuronal IFs in human. Nevertheless, as for human fibroblasts, there was no report of cell death in iPSC-motor neurons (MNs), limiting the relevance of these models for the investigation of the cytoskeleton deficits in patients.

Neurodegeneration has been investigated in primary neurons derived from independent GAN knock-out mouse (see next paragraph). An initial study, performed on GAN^–/–^ cortical neurons reported a massive degeneration (90%) at 15 days in culture (Allen et al., 2005), which would have been extremely robust if the same group did not contradict himself. Thus, similar pictures (showing DAPI staining with decreased tubulin staining) were simultaneously considered as dead cells (Allen et al., 2005), and alive cells with decreased tubulin content (Wang et al., 2005), to fit with a statement on microtubules. Independently, quantification of cell number over time revealed the modest degeneration of GAN^–/–^ cortical neurons from 15 days, a time where wild-type cells also exhibit some degeneration (Scrivo et al., 2019).

Neurons from the dorsal root ganglia (DRG) of another GAN model were characterized (Israeli et al., 2016). While they do not exhibit cell death in normal culture conditions, degeneration occurs when metabolic stress is applied (galactose and inhibitor of mitochondrial functions), hence revealing a susceptibility of GAN neurons towards degeneration. Giant Axonal Neuropathy DRG neurons also induce aggregation of all neuronal IFs, including neurofilament light (NFL), medium (NFM), heavy (NFH) subunits, peripherin and α-internexin (illustration no. 4 in Figure 6B). Similarly, GAN cortical and motor neurons exhibit aggregation of neuronal IF proteins (illustration no. 3 in Figure 6B) (Bomont, personal data). Altogether, these studies place these diverse primary neuronal cells as valuable tools to study IF biology and the specific process underlying susceptibility to neurodegeneration. An interesting avenue is mitochondria, as motility and bioenergetic defects have been evidenced in primary fibroblasts of patients and in GAN DRG neurons (Israeli et al., 2016; Lowery et al., 2016). The role of the metabolic balance in GAN pathogenicity is an exciting area to pursue, and recent work indicates the importance of glycosylation sites on Gigaxonin to modulate its activity on IFs (Chen et al., 2020). Identifying the metabolic conditions that directly control Gigaxonin activity and modulate GAN phenotypes would be particularly important to precise the mode of regulation of this ubiquitous E3 ligase and may explain why, among others, neuronal tissues are most severely affected.

Study of the recessive GAN pathology has been conducted in three independent mouse models, though the deletion of the promotor-exon1 (GAN^Δ*ex*1^, called GANJPJ in illustration no. 7 in Figure 6B) and early exons (GAN1^Δ*ex*3–5^ for GANYY and GAN2^Δ*ex*3–5^ for GANPB) leading to a premature stop codon. The first mouse to be described, the GAN1^Δ*ex*3–5^ was presented as recapitulating the human pathology (Ding et al., 2006), with a deterioration of motor functions from 6 months of age accompanied by axonal loss and densely packed neurofilaments. Unfortunately, the reported defects were not quantified in the original publication and appropriate behavioral tests, performed independently did not confirm the neurological symptoms in this mouse (Ganay et al., 2011). The two other GAN mice (GAN^Δ*ex*1^ and GAN2^Δ*ex*3–5^) presented both a mild phenotype with no overt neurodegeneration (Dequen et al., 2008; Ganay et al., 2011). In the GAN^Δ*ex*1^ mouse, the 10% increase of muscle denervation and 27% decrease in axon caliber, observed from 6 months of age did not induce spinal motor neuron degeneration nor affected motor performance over a 15 month-period (Dequen et al., 2008). Analyzed over a longer period (18 months) and with a large set of motor and sensory behavioral tests, the GAN2^Δ*ex*3–5^ mouse was shown to develop a late onset phenotype, with no axonal and motor neuron loss (Ganay et al., 2011). Interestingly, GAN2^Δ*ex*3–5^ were backcrossed in two different pure backgrounds and revealed an exclusive penetrance of the phenotype, with alterations of (i) motor performances solely in 129/SvJ mice from 15 months of age, and (ii) sensitivity from 12 months of age in C57BL/6 mice. These findings reveal the existence of modifiers genes for GAN that modulate specific functions of Gigaxonin in the motor and sensory systems. In addition to a late onset, the phenotype was quite mild, in comparison to the total loss of sensitivity and motility seen in patients during adolescence. Nonetheless, both GAN^Δ*ex*1^ and GAN2^Δ*ex*3–5^ mice exhibit the characteristic aggregation of IFs throughout the nervous system. Starting at the early age of 3 months, the steady levels of IF proteins (NFL, NFM, NFH, α-internexin, and vimentin) are increased in the brain, cerebellum and spinal cord of the GAN^Δ*ex*1^ mice (Dequen et al., 2008). A consistent increase in the abundance of NF proteins has been evidenced in the three mouse models in the brain, spinal cord and sciatic nerves, escalating to a seven fold increase for NFL in brain at 12 months (Ganay et al., 2011). At this age, ultrastructural analysis revealed an abnormal spatial distribution of the NF network, together with an increase in NF diameter, although not as severe as what is described in patients (illustration no. 6 in Figure 6B). In particular, none of the models revealed the characteristic presence of giant axons filled with densely packed NFs. In the brain, neuronal IF inclusions have been identified in the cerebral cortex and thalamus (NFH and α-internexin) of GAN^Δ*ex*1^ mice (illustration no. 7 in Figure 6B) (Dequen et al., 2008). These data show that detection of NF abnormalities (from 3 months) occurs prior to expression of mild symptoms (12 months), leading to the hypothesis that NF may not be a causal factor for neurological deficits, unless NF defects might be too mild to induce neurodegeneration in the present mouse models.

Thus, it is now established that the strategy of knocking-out the murine *GAN* gene does not phenocopy the severity of the human pathology. This phenomenon observed for numerous diseases is recognized as a genetic compensation response (GCR) in which upregulation of related genes contribute in maintaining functions in the context of deleterious mutations. This mechanism has been recently elucidated in both mouse and zebrafish species. Thus, in the presence of a premature stop codon on the mRNA (as expected in the GAN^Δ*ex*3–5^ mice), the machinery leading to its degradation (called nonsense-mediated mRNA decay) triggers a specific signaling response to upregulate the transcription of related genes (El-Brolosy et al., 2019; Ma et al., 2019). This novel mechanism, called nonsense-induced transcriptional compensation (NITC) may be gene-dependent but explains why many knock-out strategies fail in mouse.

Considering the challenges in modeling the GAN pathology in mouse, a novel strategy was initiated on an alternative vertebrate model, the zebrafish or *Danio rerio*. This species presents many advantages to study the nervous system at a physiological level. First, zebrafish presents a high conservation of the nervous system and genes (>70%) with human. Second, the fertilized eggs are produced *ex vivo*. This allows various manipulations from one cell-stage, including gene knock-down (for recessive diseases), mRNA overexpression (for dominant diseases or rescue experiment) using microinjection and screening of libraries of chemicals by simple dilution in the water. Third, the high number of eggs generated per clutch provides substantial statistical power to experimental outcome. Fourth, the optic transparency of embryos permits to investigate neuronal and neuromuscular integrity at the physiological level, within tissues. Thus, the *GAN* orthologous gene, identified at one copy on the zebrafish genome was cloned and characterized. The zebrafish Gigaxonin (z-Gigaxonin) shows a 78% identity with the human Gigaxonin and shares the same domain organization with the BTB motif in N-terminus and six Kelch repeats at the C-terminus (Arribat et al., 2019). The spatial and temporal analysis of the *gan* mRNA transcripts revealed ubiquitous expression and distribution, in agreement with the data gathered on the human Gigaxonin (Bomont et al., 2000). Repression of z-Gigaxonin was first performed in a transient set-up, by injecting *gan* antisense morpholino oligonucleotides (MO) in fertilized eggs to generate *gan* morphants. In the zebrafish community, this methodology, which impairs pre-mRNA splicing and blocks translation is widely used to silence gene expression. The discovery of the CRISPR technology, enabling the creation of stable KO lines evidenced that several mutants failed to reproduce the phenotypes described in the corresponding morphants (Kok et al., 2015). This poor correlation between morpholino-induced and mutant phenotypes in zebrafish questioned the specificity of the MO approach and suggested off-targets effects. Reciprocally, the comparison of the transcriptome and proteome between MO and KO lines altering the same gene revealed a specific genetic compensation in the knock-out line (Rossi et al., 2015), which constituted the basis of the discovery of the NITC pathway that can be triggered in KO design (El-Brolosy et al., 2019; Ma et al., 2019). In this respect, the MO approach is more relevant to study gene functions. For GAN, the specificity of action of *gan* morphants was demonstrated by the analysis of oligonucleotides targeting two independent regions of the *gan* mRNA, dose-dependent curves, use of mismatch counterparts and rescue using the human *GAN* mRNA. Thus, repression of z-Gigaxonin induces severe neurological phenotypes, including the shortening/absence of axons of primary spinal motor neurons [pMNs, first born around 1dpf (day postfertilization)], their abnormal protrusion from the spinal cord, and a global abolishment of neuromuscular junctions (illustration no. 8 in Figure 6B). Intriguingly, this was also accompanied by a loss of secondary motor neurons (sMNs, born around 2dpf), which was not resulting from cell death but from incapacity to generate novel neurons. The mechanism underlying this unexpected outcome is discussed in the next section. Altogether, these neurological alterations induce a severe locomotor defect, which is also significant in the genetic line created through the knocking-out of the entire gene, yet less penetrant. Thus, swimming defects upon touch stimuli are observed in 72.4% of the morphants (49% of mutants) at 72 hpf (hours postfertilization) and persist latter on freely moving assay, with 80% of immobile morphants/mutants at 5 dpf. Considering the design of the mutant, which generates premature stop codon within exon 1, this modest discrepancy is most probably due to the activation of the NITC pathway in the genetic line. For GAN, both methodologies of gene inactivation demonstrate that absence of Gigaxonin causes severe neuronal alterations resulting in locomotor impairment.

In conclusion, several cellular and mouse animal models have been generated for GAN. There were very valuable and complementary to study of IF aggregation within and outside the nervous system and to decipher the pivotal role of Gigaxonin in IF biology. On the other hand, the neurodegenerative traits of GAN could not be reproduced. Neuronal loss was only evidenced in vitro on aging GAN neurons or upon stress, and motor and sensory deficits are mild and of late onset in the GAN mouse. Mimicking the loss of motility seen in young GAN patients, the *gan* zebrafish represents the first robust animal model for the pathology.

## Gigaxonin Functions

**FIGURE 7:**
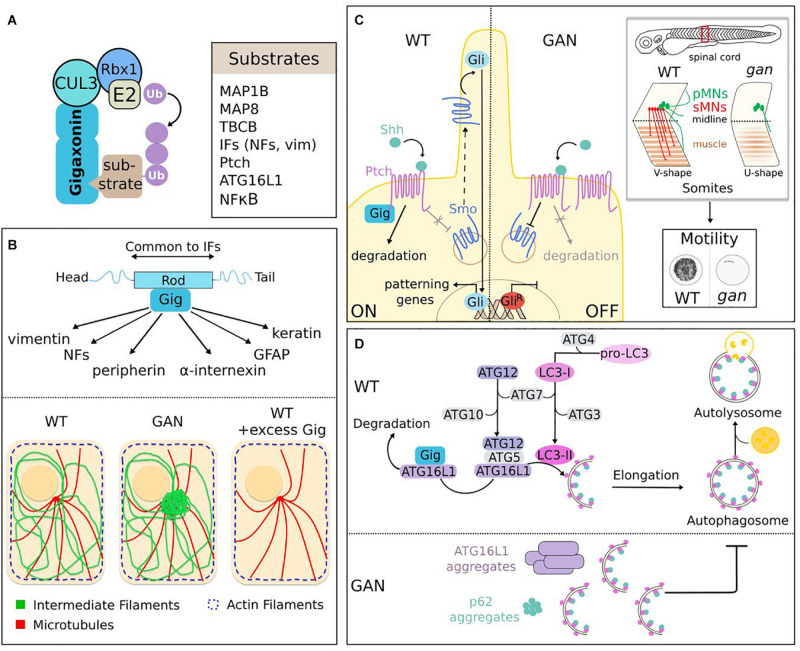
Functions of the Gigaxonin-E3 ligase. **(A)** Identified substrates for Gigaxonin include cytoskeletal components (MAPs, TBCB, IFs), Shh receptor (Ptch), autophagy core protein (ATG16L1) and transcriptional factor (NF_K_B). **(B)** Gigaxonin controls the turn-over of the IF family. Through interaction with the central α-helical rod domain, a common domain of IF proteins, Gigaxonin controls the steady-level of IF family, including neuronal (NFs, peripherin, and α-internexin) and non-neuronal (vimentin, GFAP, and keratin) IF types. This is mediated by Gigaxonin-dependent ubiquitination (as revealed for peripherin). In absence of Gigaxonin (patients and GAN models), IF proteins bundle at perinuclear region, while actin and microtubules do not show overt alteration. Excess of Gigaxonin completely destroys IFs, including bundles in GAN samples. **(C)** Control of the initiation of Shh signaling by Gigaxonin is essential for locomotion. In normal condition (left), Gigaxonin initiates signal transduction by degrading Shh-bound Ptch receptor, hence suppressing its constitutive inhibition on the signal transducer Smo, allowing its translocation to the primary cilium, and leading to the transcriptional activation of Gli. In absence of Gigaxonin (right), the repression of Smo is maintained by accumulated Ptch receptor, which hampers the initiation of Shh signaling and favors the generation of a repressor form of Gli. In zebrafish, this defective Shh signaling impairs motility of *gan* larvae (cumulative representation of the spontaneous locomotion of larvae for 1 h). This severe motility defect is caused by a dramatic remodeling of spinal cord architecture, [shortening of pMNs axons (primary motor neurons, green), defective production of sMNs (secondary motor neurons, red)], and failure to generate proper neuromuscular junctions (NMJ). This phenotype is accompanied by a muscular defect (neuronal-independent or dependent), characterized by “U-shape” myofibers instead of the normal “V-shape.” **(D)** Gigaxonin regulates autophagosome production. Elongation of phagophore membrane involves two ubiquitin-like conjugation systems: ATG12 and LC3 undergo transfer from E1 (ATG7) to E2 (ATG10 and ATG3) enzymes. ATG12-ATG5 subsequently forms a complex with ATG16L1, which addresses it to the nascent membrane to act as an E3 ligase for the lipidation of LC3, to generate LC3-II. Gigaxonin induces the ubiquitin-dependent degradation of ATG16L1, and its absence in GAN neurons causes a failure to generate productive autophagosomes upon autophagy induction. As a result, ATG16L1 bundles in GAN neurons, the autophagy receptor P62 accumulates and autophagosome-lysosome fusion is impaired, hence blocking autophagy flux.

So far, seven substrates or family of substrates (IF family) have been identified for Gigaxonin (Figure 7A). They include proteins involved in cytoskeleton architecture (MAP1B, MAP8, TBCB, and IFs), neuronal specification (Ptch) and autophagy (ATG16L1). While we will focus here on the pathways implicated in neurological physiology, Gigaxonin was also shown to modulate chemosensitivity of cancer, through ubiquitination of NFκB (Veena et al., 2014).

### Gigaxonin Is a Universal Regulator of the Intermediate Filament Cytoskeleton

Intermediate Filament regulation is the best-known function of Gigaxonin. Evidenced 2 years after the discovery of the GAN pathology (Pena, 1981), the aggregation of vimentin in patient-derived fibroblasts constitutes a key cellular tracer that paved the avenue for functional studies, in spite of the fact that it is non-neuronal. From a cell biology point of view, vimentin and fibroblasts are relevant to study Gigaxonin functions, considering the wide impact of GAN on IF architecture beyond neuronal tissues. As discussed earlier, the detection of densely packed NFs within giant axons in the nervous system has been extended to various classes of IFs in patient tissues (Figure 3). The development of cellular and animal models for GAN (Figure 6) has further completed the picture of a general and massive collapse of cytoplasmic IFs that constitute the nervous system, hair, muscle, blood vessel/mesenchyme and skin (Figure 7B). In the nervous system, Gigaxonin depletion affects neuronal IFs in their spatial orientation, distribution and abundance (see previous section for details). Thus, besides nestin, which has not been investigated yet, all neuronal IF proteins are abnormally bundled in absence of Gigaxonin: NFL, NFM, NFH, α-internexin, and peripherin. While this aggregation could results from a qualitative effect on distribution, several studies have shown an increase of neuronal IF levels in GAN iPSc-derived neurons, DRG neurons and mouse tissues (Dequen et al., 2008; Ganay et al., 2011; Johnson-Kerner et al., 2015a; Israeli et al., 2016). Interestingly, no overt change in vimentin level could be evidenced in human fibroblasts, MEFs and iPS-derived motor neurons (Mahammad et al., 2013; Johnson-Kerner et al., 2015a). Still, vimentin abundance is regulated by Gigaxonin, as its prolonged overexpression is sufficient to induce complete destruction in control cells after 72 h (Figure 7B). This effect is so robust that it also enables the clearance of vimentin bundles in fibroblasts from patients and GAN mouse embryonic fibroblasts (Mahammad et al., 2013). From this initial key findings, the potent effect of Gigaxonin has been extended to various IFs of the nervous system, and is now confirmed in normal cells [neurons in Mahammad et al. (2013), Israeli et al. (2016) and astrocytes in Lin et al. (2016)], but also in disease context [GAN iPSC-derived neurons in Johnson-Kerner et al. (2015a), and GAN DRG in Israeli et al. (2016)]. To understand this unique action on multiple IFs proteins, that constitute the cytoskeleton of tissues throughout the body, it is important to introduce the structure of IF proteins. IF proteins have a tripartite organization, with a central α-helical rod domain flanked by non-α-helical domains at the extremities, also called the head and a tail domains (Figure 7B). While the extremities show variation in sequence and length, the central rod domain is highly conserved across IF proteins. This is of particular importance, as this central domain constitutes the structural backbone enabling the sequential steps necessary to assemble mature IFs across IF types (Herrmann and Aebi, 2016). In agreement with the structure of IFs, and the control of multiple IF types by Gigaxonin, the later has been shown to specifically interact with the rod domain of vimentin (Mahammad et al., 2013). This interaction seems to be direct, as assessed by ELISA assay using recombinant vimentin proteins. Accordingly, additional studies showed that other IF types can be found in Gigaxonin immunocomplexes in HEK cells and astrocytes (Johnson-Kerner et al., 2015b; Lin et al., 2016). Interestingly, the potent action of Gigaxonin has been confirmed for keratin and GFAP, mutated and aggregating, respectively in the skin disease EBS and Alexander disease (Lin et al., 2016; Buchau et al., 2018). Gigaxonin was shown to clear GFAP network in swi13 stably expressing various mutants but resistance was conferred to some GFAP mutants, which exhibit reduced binding capacity to Gigaxonin. While these mutations lies within the rod domain, the screening of additional mutants in Alexander diseases will be important to ascertain this hypothesis and map the interaction motif with Gigaxonin. While the interaction of Gigaxonin with IF proteins has been repeatedly confirmed, there was challenges in demonstrating its mode of action on IFs. Indeed, only a partial recovery of both vimentin and GFAP levels upon Gigaxonin overexpression was seen with proteasome inhibitor, and laddering of vimentin could not be evidenced with in vivo and in vitro assays, and very low with GFAP (Mahammad et al., 2013; Lin et al., 2016). One study was able to show a Gigaxonin-dependent ubiquitin laddering of peripherin in GAN DRG neurons (Israeli et al., 2016). Interestingly, authors also showed that DRG neurons depleted in Cul3 exhibit increased levels and aggregation of all neuronal IF proteins, similarly to GAN. Now, more work is needed to further define the interacting motifs on IFs, the identity of the ubiquitin chains, and mode of regulation of Gigaxonin on this stable but dynamic cytoskeletal network.

Altogether, the complementarity of several laboratories, and the focus on non-neuronal primary fibroblasts have established that Gigaxonin is the first factor controlling the degradation of the IF family (Bomont, 2016). This has a major impact on cell biology as no drug or compound exists to clear IFs (as it is largely developed for microtubules and actin). Moreover, considering the >80 different diseases caused by mutations in many IF types, Gigaxonin represents one exciting candidate for therapeutic perspectives for human health.

### What About Microtubules?

Two microtubule-associated proteins and one chaperone of tubulin have been identified as interacting partners for Gigaxonin: MAP1B (Allen et al., 2005), MAP8 (Ding et al., 2006), and TBCB (Wang et al., 2005) (see Box 1). The abundance of MAP1B, MAP8, and TBCB is decreased upon Gigaxonin overexpression. While this degradation is fully restored upon proteasome inhibition, the Gigaxonin-dependent ubiquitination has only been firmly evidenced for TBCB. Interestingly, two Gigaxonin disease mutants were shown to interact with TBCB, and one was equally promoting TBCB ubiquitination, as efficiently as the wild-type Gigaxonin. This emphasizes what we discussed earlier regarding the cautiousness in the interpretation using Gigaxonin mutants in overexpression system, as they can retain activity. Nevertheless, TBCB level was shown to be unaffected between control and patient fibroblasts, and by overexpression of Gigaxonin (Cleveland et al., 2009; Mahammad et al., 2013); and MAP1B was shown to be equally abundant in control and GAN iPSC-derived motor neurons (Johnson-Kerner et al., 2015a).

**BOX 1:**
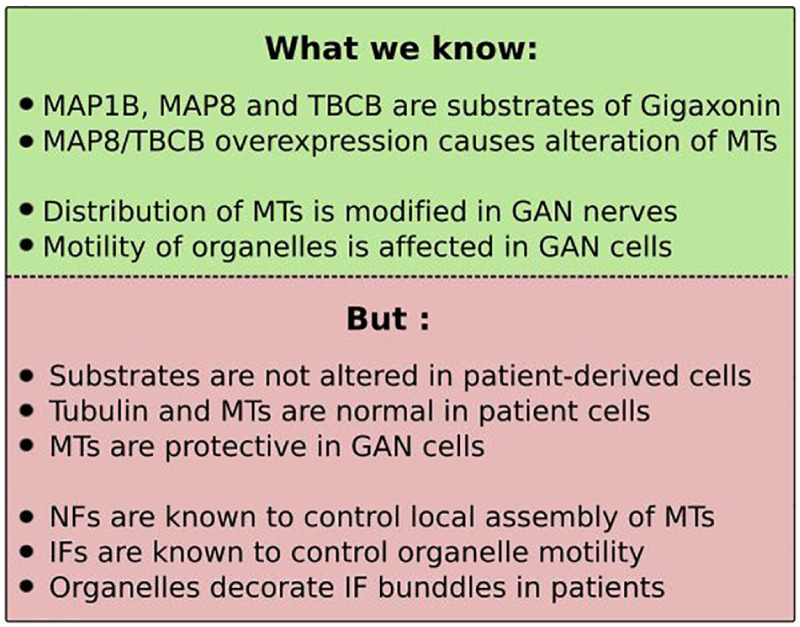
Role of microtubules in GAN? Caution.

In light of the microtubule-binding capacities of the substrates, claims have been made to link microtubules (MTs) to GAN deficits and to convince on their relevance for the pathology, but these suffered from lack of reproducibility in independent studies and incomplete assessment (see Box 1).

MAP8 overexpression was shown to cause abnormal MT organization ranging from wavy pattern to aggregation, trapping dynein motor proteins (Ding et al., 2006). TBCB overexpression was found to clear MTs, and Gigaxonin addition to reverse this, but no quantification was provided (Wang et al., 2005). Instead, in another study, the effect of TBCB on MTs was shown to be modest, with only 36% of cells presenting, at most a slight reduction in MT intensity (Cleveland et al., 2009). With this partial knowledge, concluding that these substrates recapitulate the GAN pathology is highly hypothetical. Particularly, examination of MTs or tubulin levels has not revealed overt alterations in none of the patient derived cells (Bomont and Koenig, 2003; Cleveland et al., 2009; Mahammad et al., 2013; Johnson-Kerner et al., 2015a). In contrary to what is proposed, the fact that MTs have been found sparser in peripheral GAN nerves (Ganay et al., 2011) doesn’t imply a direct action by MTs. First, tubulin levels are equal in WT and KO tissues (Dequen et al., 2008; Ganay et al., 2011). Second, data coming from patients suggest a change in the distribution of MTs along the nerves. Indeed, in GAN axons, MTs are excluded from densely packed NFs and form clusters, and many axons devoid of NFs are filled by MTs (Donaghy et al., 1988; Treiber-Held et al., 1994). This dependence towards NF density has been evidenced in several animal models and is mediated by the ability of specific motifs in NF (and IF) proteins to bind to soluble tubulin, hence modulating the local assembly of MTs (Bocquet et al., 2009). In this regard, MT disorganization could be caused by NF aggregation. Third, the alteration of the motility of vesicles (Ding et al., 2006), and mitochondria in GAN DRG neurons and patient fibroblasts (Israeli et al., 2016; Lowery et al., 2016) is not necessarily caused by MT putative dysfunction. As MTs, IFs are major regulators of vesicle trafficking by tethering to organelles and may be at play in GAN, as proposed by the alignment of mitochondria along bundled vimentin in GAN fibroblasts (Lowery et al., 2016). Fourth, the suggestion of the causal role of MT alteration in IF aggregation is not supported in GAN primary fibroblasts. Indeed, not only TBCB abundance is not altered in patient cells, MTs clearance (upon overexpression of TBCB or the potent MT destabilizing agent TBCE, or nocodazole) does not produce vimentin aggregation in control fibroblasts. Instead, MTs was found to be protective in patients cells, from a greater collapse of vimentin upon nocodazole treatment (Cleveland et al., 2009). While a contribution of MT is not excluded, its demonstration requires further work.

Another concern is the relevance of MT-related substrates for neurodegeneration in GAN. Individually, MAP1B and MAP8 were shown to be sufficient to cause death when overexpressed in wild-type neurons, and to substantially improve survival rate when silenced in GAN neurons (Allen et al., 2005; Ding et al., 2006). These conclusions can be questioned, considering that, as mentioned in the previous section, measure of cell death was based on tubulin staining and that no wild-type controls were added to estimate the magnitude of the rescue of cell death in silencing experiment. Moreover, the 1.5–3 fold increase in the levels of MAP1B, MAP8, and TBCB in KO tissues does not constitute by itself an argument for their role in neurodegeneration.

In conclusion, Gigaxonin controls important players of MT dynamics but the statement of their causal role, and of MT implication in the pathogenesis of GAN is an incorrect shortcut with our current knowledge. Additional work should definitively be performed to deepen this and identify possible roles of MTs in GAN.

While the discovery of the pivotal role of Gigaxonin in controlling IF cytoskeleton was directed by its aggregation in patients, novel roles have recently unexpectedly emerged from exploration of the biological models of GAN.

### Gigaxonin Controls Shh Signaling Pathway to Sustain Locomotor Activity

As discussed in the previous section, the *gan* zebrafish constitutes the first robust animal model for the pathology (Arribat et al., 2019). Indeed, inactivation of the z-Gigaxonin was shown to abolish motility in 80% of both morphants and mutants, hence mimicking the loss of motility seen in patients (Figure 6). Alteration of the motor system was further detailed in three-dimensional imaging to reveal a dramatic remodeling of spinal cord architecture, with absence of many axons, the remaining being shortened and abnormally protruding from the spinal axis, and with lack of neuromuscular junctions. The deciphering of the underlying mechanisms (Figure 7C) was guided by interesting observation on the alteration of muscle architecture. Indeed, in absence of z-Gigaxonin, the structure of somites is damaged and presents abnormal sarcomeric organization. Myofibers are less dense and somite boundaries define a “U-shape” instead of the normal “V-shape.” Specifically, this characteristic is observed in mutants of the Sonic Hedgehog (Shh) pathway (Chen et al., 2001; Varga et al., 2001), one of the key developmental machinery that assigns neuronal (Jessell, 2000) and muscle (Te and Reggiani, 2002) fate in vertebrates. Implicated during embryogenesis but also in homeostasis in adults, Shh impairment causes a wide range of human diseases, ranging from malformations of the nervous system, of the axial skeleton and limbs to cancer (Bale, 2002). Shh signaling is triggered by the active fragment of the morphogen Shh, whose binding to the Ptch receptor in progenitor cells alleviates the repression of another receptor Smo, therefore inducing a cascade of events leading to the transcriptional activation of specifying genes (Lee et al., 2016) (Figure 7C). Thus, concomitantly to neurodegeneration, birth of MNs was investigated in the *gan* zebrafish model. While cell death was not evidenced within spinal motor neurons, an overt reduction in their production was shown in both morphants and mutants. In zebrafish, spinal motor neurons are generated in two successive waves (around 24 and 48 hpf), and only the second is impaired in *gan* zebrafish, leading to a total absence of secondary axons. On the contrary, primary MNs are generated but they project truncated and misguided axons, as mentioned above in three-dimensional imaging. Thus, these data revealed a differential and temporal effect, causing axonal abnormalities in the first wave, and an improper specification of MNs in the second wave, as further demonstrated by the down-regulation of Shh-responsive genes. Altogether, the different neuronal defects (and possibly muscle alterations) in *gan* zebrafish impede nerve conductivity through loss of neuromuscular junctions that is sufficient to abolish locomotion. Considering the known roles of Shh signaling in neuromuscular specification, but also in axonal guidance (Aviles et al., 2013), and the data presented above, its implication in *gan* phenotypes has been investigated using drugs that modulate its activity. Thus, inhibition of Shh signaling (using cyclopamine) during the second wave in control embryos reproduces the *gan* phenotype, with “U-shape” somites and absence of secondary MNs. Conversely, increase of Shh activity (using purmorphamine) restores “V-shape” somites in 24% of morphants. The recovery of MN specification was obtained in *gan* treated embryos, but penetrance increases from 17.5 to 70% when drug was applied earlier (during the first wave), suggesting a Shh-dependent requirement of early event for sMN specification. Analysis of distinct biological models in zebrafish, mouse, and human demonstrated that Gigaxonin acts as a positive regulator of Shh. Thus, in situ hybridization revealed a decrease of Shh-responsive gene expression in *gan* morphants and mutants. In addition, repression of Gigaxonin in the Shh Light-2 reporter line impairs its responsiveness to the morphogen. Finally, translocation of Smo to the cilium, which represents an important readout of Shh activation is severely impaired in two independent GAN fibroblasts exposed to Shh, hence evidencing a decrease responsiveness to Shh in human cells. Deciphering the molecular mechanisms by which Gigaxonin controls Shh signaling would have been ideal in drosophila species, a powerful genetic system that is widely used in this field. Nevertheless, Gigaxonin is not conserved in fly, which may suggest that Gigaxonin participates in the evolution of the system in vertebrates (Briscoe and Therond, 2013). Thus, the underlying molecular mechanism by which Gigaxonin positively regulates Shh signaling has been deciphered in cell lines. It was shown that Gigaxonin interacts with the receptor Ptch and induces its degradation in a Shh-dependent manner, which suggests that the E3 ligase controls the entry point of Shh signaling, and not the basal recycling of the receptor, as reported for other E3 ligases Itchy and Smurf (Huang et al., 2013; Chen et al., 2014). Interestingly, ubiquitination of Ptch was not detected in presence of Gigaxonin but was considerably enriched in Gig-immunocomplexes, suggesting that a small pool of Ptch is modified by Gigaxonin within a cell. Certainly, further work is needed to deepen this ubiquitination aspect and the regulation of Gigaxonin interaction. Monitoring simultaneously Ptch activation and Gigaxonin interaction upon Shh stimulation would help understanding the temporal and spatial mode of action of the E3 ligase in physiology.

Importantly, this work provides the first hints for a developmental origin in GAN, and adds to relevant findings of a developmental contribution in the setting of post-natal neurological diseases. Indeed, emerging evidence from patients and animal models suggests that abnormal neurodevelopment is a component of the pathophysiology of adult neurological diseases, including Alzheimer’s and Huntington’s diseases. The most striking demonstration is that mice expressing mutant huntingtin solely during developmental stage recapitulate symptoms of the human pathology (Molero et al., 2016). For human GAN, this hypothesis is supported by atrophies in various regions of the nervous system using MRI (magnetic resonance imaging), and the presence of a morphological marker of developmental deficits in patients (Demir et al., 2005). Demonstrating the role of Gigaxonin-mediated Shh activity in sustaining motility in zebrafish, this initial study opens a novel avenue for understanding the origin of GAN pathophysiology. Future work will determine whether inhibition of Shh signaling contributes or is sufficient to induce neuronal loss and neurological deficits in patients. Finally, considering the role of the impaired Shh signaling in various diseases, Gigaxonin may represent one interesting target to modulate Shh response as potential therapy for other conditions.

### Gigaxonin Regulates Autophagosome Production

As many neurodegenerative diseases, autophagy is altered in GAN but deeper exploration revealed that Gigaxonin controls an essential step of the pathway, by driving membrane expansion of the phagophore to produce mature autophagosomes that can fuse to lysosomes (Scrivo et al., 2019) (Figure 7D). This key process was evidenced in GAN primary cortical neurons in which lipidation of the LC3 protein (LC3-II), occurring at the site of membrane elongation was monitored under basal and experimental conditions. Using conventional drugs to boost autophagy and block fusion of autophagosomes with lysosomes, a specific defect in the net production of LC3-II (presumably autophagosomes) was shown in GAN neurons. More precisely, GAN neurons generate autophagosomes but are in incapacity of increasing production over prolonged induction of autophagy. Studies of additional markers of autophagy flux converged towards an inhibition of the elongation step, leading to an accumulation of the main autophagy receptor p62, and decrease detection of autophagosome-lysosome events. LC3 lipidation is a tightly regulated process that involves two conserved ubiquitin-like conjugation systems. Structurally related to ubiquitin, the ATG12 and LC3 proteins are transferred by E1- and E2-like enzymes to their substrates ATG5 and ATG3 (Figure 7D). Subsequently, ATG12–ATG5 acts as the E3 ligase enabling lipidation of LC3. In this complex hierarchical process, Gigaxonin was shown to regulate the ATG16 protein (Scrivo et al., 2019), which binds ATG12–ATG5 and addresses it onto nascent membrane (Fujita et al., 2008), hence specifying the site of lipidation. Crucial for autophagosome formation, ATG16L1 is essential for survival and ATG16L1 KO mouse is lethal 1 day after birth (Saitoh et al., 2008). Gigaxonin binds to the C-terminal WD40 domain of ATG16L1 and induces its degradation. As for IF proteins, the clearance of ATG16L1 by ectopic Gigaxonin is extremely robust and is only modestly rescued upon proteasome or autophagy inhibition. This constituted an obstacle to investigate ATG16L1 ubiquitination, but was solved by evidencing its decrease using Gig siRNA. Single-cell analysis completed the picture and demonstrated co-labeling of K48-ubiquitin chains onto ATG16L1 when Gigaxonin was present. The abnormal bundling of ATG16L1 in the soma of GAN neurons further confirmed its regulation by Gigaxonin, and specificity towards ATG16L1 was provided by rescue experiment using lentiviral expression of the E3 ligase. Equally efficient, Gigaxonin clears aggregates in GAN neurons and leads to the destruction of ATG16L1 in control neurons. Altogether, this initial study unveils a key regulatory mechanism that drives the dynamic of autophagosome production. In absence of Gigaxonin, accumulated ATG16L1 would impair membrane elongation and cause the stacking of phagophores/incomplete autophagosomes, as shown upon overexpression, or deletion of ATG16 (Fujita et al., 2008; Saitoh et al., 2008). An important perspective would be to determine whether Gigaxonin regulates autophagosome production in basal or specific physiological conditions. Indeed, limitation of the study concerns the investigation of LC3 lipidation and ATG12-ATG5 docking to membrane in basal condition, which was challenged by the low expression level of LC3 and high density of dots in primary neurons. Thus, while the later represents the ideal system to investigate endogenous mechanisms, other systems using fluorescently labeled LC3 overexpression in cell lines may be preferred for some aspects. Another attractive perspective is to use the GAN model to investigate the spatial control of autophagy within neurons (Bomont, 2019). In polarized cells, the best-described process is distal: biogenesis occurs at axonal tip, phagophores elongate and autophagosomes mature and fuse to lysosomes while undergoing retrograde transport towards the soma. Still, autophagosome biogenesis has also been evidenced in the soma (Maday and Holzbaur, 2016; Stavoe and Holzbaur, 2019) and GAN might constitute a powerful biological system to study its process and function. Finally, the novel avenue of the control of ATG16L1 by Gigaxonin may offer therapeutic perspective for GAN but also for other diseases, for which the E3 ligase may represent a molecular switch to diminish autophagy activity.

## Common Mechanisms Amongst E3 Ligases: Emerging Roles in Autophagy

**FIGURE 8:**
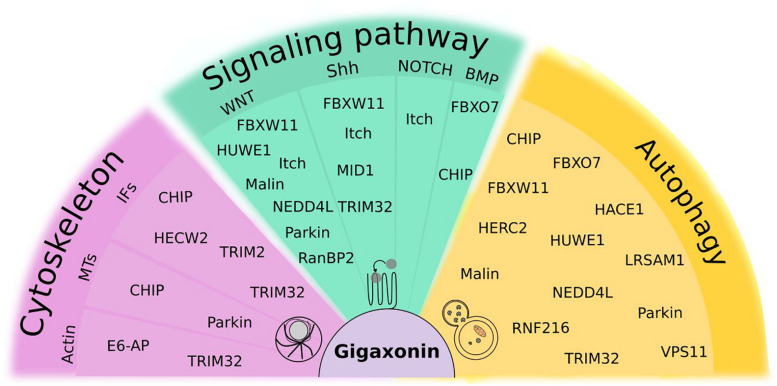
Focus on ubiquitin(like)-dependent regulation of cellular mechanisms shared by Gigaxonin and other E3 ligases. Cytoskeleton comprises actin, microtubules (MTs) and Intermediate Filaments (IFs). Signaling pathways, selected from their roles in spinal cord development and maintenance include WNT, Sonic Hedgehog (Shh), NOTCH and Bone Morphogenetic Protein (BMP). For Fibroblast Growth Factor (FGF) and Retinoic acid (RA), no function of E3 ligases was reported to our knowledge. As substantial proportion of E3 ligases (30%) control the autophagy pathway. Some E3 ligases act at multiple levels within the same pathways (like Itch, NEDD4L), and 23% of them exhibit functions in different pathways, with Gigaxonin, TRIM32, and CHIP crossing all pathways.

**FIGURE 9:**
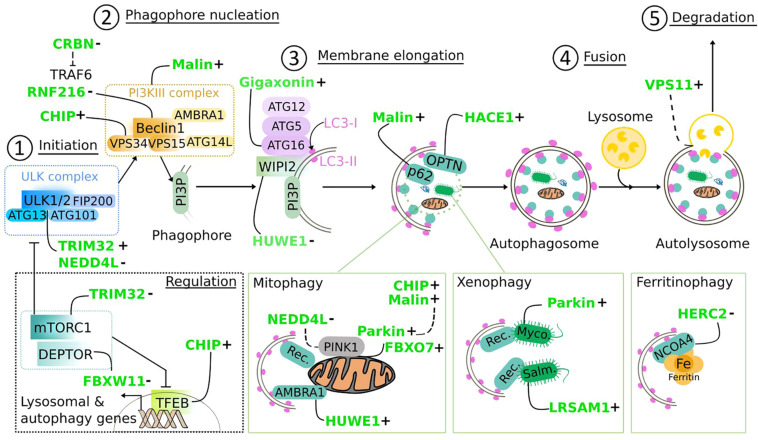
Regulation of the autophagy pathway by E3 ubiquitin ligases. E3 ligases mutated in neurodegenerative diseases (in green) target core components of the autophagy machinery, from the initiation **(1)** to the fusion of the autophagosome to lysosomes **(4)** to positively (+) or negatively (−) regulate autophagic flux. Following (macro)autophagy induction, the inhibitory effect of mTORC1 on the ULK complex is lifted, allowing the activation of the ULK1/2 kinases **(1)**, leading to a cascade of phosphorylation events that contribute to the activation of the PI3KIII complex via Beclin1, to promote PI3P synthesis on nascent phagophore **(2)**. Interacting with PI3P, WIPI2 subsequently recruits ATG16L1 in complex with ATG12-ATG5, the E3 ligase that catalyzes the lipidation of LC3 at membranes, a step **(3)** required for membrane elongation and autophagosome formation. Fusion with lysosome **(4)** leads to the degradation of sequestered material, which can be non-specific (called bulk autophagy) or specific (called selective autophagy). Selectivity is triggered by ubiquitination of substrates [**(bottom panels)** mitochondria, pathogens, and Ferritin], and mediated by adaptor proteins that direct them to autophagosomes thanks to LC3-interacting region (LIR domain) and ubiquitin-associated domain (UBA) domain. “Rec” for mitophagy: p62, OPTN, NDP52, TAX1BP1, NBR1, or AMBRA1; of note, some mitochondrial proteins have a LIR domain and contribute to mitophagy. “Rec” for xenophagy: p62, NDP52, NBR1. Myco., *Mycobacterium* tuberculosis; Salm., *Salmonella* Thyphimurium. mTOR signaling, which is an upstream inhibitor of autophagy induction is also regulated by E3 ligases: through action on mTOR inhibitors (DEPTOR) and transcriptional control of autophagy genes (TFEB). Dashed line: partial demonstration of mechanistic. E3 ligase-dependent ubiquitination can trigger degradative or non-degradative routes. Several E3 ligases act at multiple steps of the autophagy pathway and can have antagonized actions.

Beyond the nervous system, E3 ligases mutated in neurological diseases exhibit numerous biological functions, such as DNA repair, cell division and immunity and can be implicated in the pathophysiology of other human diseases among which cancer is the most important class. Here, we will present the common cellular mechanisms shared by Gigaxonin and the other E3 ligases (Figure 8), and will focus on their role in autophagy (Figure 9). Noteworthy, we will restrict our window to pathways for which substrates have been identified, and ubiquitin(like)-dependent mode of regulation (at least partially) demonstrated, therefore excluding other functions such as some transcriptional activation types.

### Control of Cytoskeleton by E3 Ligases

Very little is known regarding the control of cytoskeleton by E3 ubiquitin ligases (those selected in this review) (Figure 8). E6-AP controls the degradation of the Ephexin5, a RhoA guanine nucleotide exchange factor that remodels actin cytoskeleton to promote excitatory synapse development (Margolis et al., 2010). TRIM32 controls the ubiquitin-dependent degradation of actin filament to sustain muscle remodeling and function (Kudryashova et al., 2005). Microtubule dynamic is modulated by Parkin, in part through degradation of α and ß tubulin (Ren et al., 2003), while CHIP regulates microtubule severing through control of katanin (Yang et al., 2013) and Gigaxonin regulates the turn-over of MAPs and TBCB. Regarding IFs, the far most recognized E3 ligase is Gigaxonin, which controls the steady-state of the entire family in multiple tissues, as discussed in this review. Other E3 ligases have shown specificity towards particular IF types, including TRIM2 for Neurofilaments (Balastik et al., 2008), CHIP for keratin (Loffek et al., 2010), TRIM32 for desmin (Cohen et al., 2012), and HECW2 for laminB1 (Krishnamoorthy et al., 2018).

### Regulation of Signal Transduction by E3 Ligases

Here, we will present E3 ligases that act as Gigaxonin on Shh signaling, expanding on other main pathways for spinal cord development and maintenance (Figure 8): Wnt, FGF (Fibroblast Growth Factor), and RA (Retinoic Acid), whose signal gradients establish the antero-posterior axis of the CNS, and Shh, Wnt and BMP (Bone Morphogenetic Protein) patterning the dorso-ventral axis of the neural tube, assisted by Notch signaling. Depending on the target (receptor, mediator/regulator or effector), E3 ligases can act as a positive or negative regulator of cell signaling.

As Gigaxonin, the Itch ligase regulates the turn-over of the Ptch receptor. Nevertheless, Ptch acts in absence of Shh, showing that Itch is not dispensable for canonical Hh signaling but essential to limit the proapoptotic activity of unliganded Ptch in non-canonical forms (Chen et al., 2014). The findings of the requirement of Shh for Ptch degradation by Gigaxonin unveil the identity of the first E3 ligase controlling the entry point of Shh signaling (Arribat et al., 2019). Consistently, Itch has also been shown to regulate the degradation of Notch in a ligand-independent manner (Chastagner et al., 2008). Regarding Wnt pathway, Itch and NEDD4L regulate, respectively LRP6 and LGR5 receptors that potentiate Wnt signaling (Vijayakumar et al., 2017; Novellasdemunt et al., 2020). Downstream the receptors, central modulators of signaling are targeted by E3 ligases. The best know is Dishevelled (Dvl) which activates Wnt cascade upon binding of Wnt to the receptor Frizzled. Thus, Dvl turn-over is regulated by Itch, Malin and NEDD4L (Sharma et al., 2012; Wei et al., 2012; Ding et al., 2013). E3 ligases are also modulating pathways through the effectors of signal transduction. Gli1 and Gli2, two transcriptional mediators of Hh response are degraded, respectively by TRIM32, Itch, and FBXW11 (also known as ß-TRCP2; Bhatia et al., 2006; Di Marcotullio et al., 2011; Wang et al., 2020); and Smad transcription factors are degraded by CHIP to modulate BMP signaling (Li L. et al., 2004). Transcription of Wnt target genes is ensured by the nuclear translocation of ß-catenin, which binds to the TCF factor. Both are targeted by E3 ligases: FBXW11 and Parkin regulates ß-catenin (Fuchs et al., 1999; Rawal et al., 2009), while RanBP2 modify TCF (by sumoylation) to promote its interaction with ß-catenin and strengthen Wnt signaling, by increasing their nuclear import and transcriptional activity (Shitashige et al., 2008). Other examples of a non-degradative regulation of cell signaling are coming with HUWE1 (de Groot et al., 2014), Itch (Infante et al., 2018), MID1 (Schweiger et al., 2014), and FBXO7 (Kang and Chung, 2015), whose ubiquitinating action on Dvl, SuFu and Fu (two regulators in Shh signaling) and RNAGE (modulator of non-canonical BMP pathway) regulates multimerization, cleavage and complex formation, which is essential for signal transduction.

Thus, E3 ligases mutated in neurological disorders control essential players of cell signaling, and they can act at multiple levels in the same pathway (like NEDD4L) and even multiples pathways (Wnt, Shh, Notch for Itch). Future investigations will probably increase this picture and further detail the elaborate mechanistic actions E3 ligases have on cell signaling. Considering that pathways also communicate between each other, mutation in a given E3 ligase may affect the equilibrium in many direct and indirect ways, hence representing a challenge when it comes to understand and treat pathophysiology in disease.

### Emerging Roles of E3 Ligases in the Control of Autophagy

Proteolysis, and in particular autophagy has been shown to be impaired in most neurodegenerative diseases. While this alteration can be indirect as a result of dysfunction of the neuron, we will discuss here the growing evidence culminating the last 2 years of the direct role of E3 ligases in regulating the autophagy pathway.

As shown in Figure 9, the E3 ligases mutated in neurological diseases target core components of the autophagy machinery, from the initiation to the fusion of the autophagosome to lysosomes. Autophagy is initiated by the activation of the ULK1/2 kinase within the ULK complex, which subsequently contributes to the activation of the PI3KIII complex (though Beclin1) that drives the formation of the phagophore, via the synthesis of a specific pool of PI3P on the primary membrane. In the initiation step, ULK1 activity is either promoted by TRIM32 through K63 chain ubiquitination (Di Rienzo et al., 2019), or silenced by NEDD4L through unusual K27/29 ubiquitin-dependent degradation following few hours of induction, as a regulatory mechanism to avoid damages from overactivity (Nazio et al., 2016). Promoting phagophore formation, Malin (in complex with Laforin) and CHN-1 (the *Caenorhabditis elegans* homologous of CHIP) activates the PI3KC3 complex, trough K63-ubiquitination of Beclin1 and several regulators (Sanchez-Martin et al., 2020) and VPS34 (Liu et al., 2018), respectively. On the contrary, RNF216 acts as a negative regulator of autophagy by targeting Beclin1 to degradation (Xu et al., 2014). Indirectly, autophagy can be down-regulated by CRBN which inhibits the ubiquitination of Beclin1 by the E3 ligase TRAF6 (Kim et al., 2019), but the mechanisms remains to be identified. Following the early steps of autophagy induction, which is largely coordinated by a myriad of phosphorylation events by core autophagy proteins (Botti-Millet et al., 2016), autophagosome is formed thanks to coordinated rounds of lipidation. Indeed, membrane elongation is powered by two ubiquitin-like conjugation systems (ATG12 and LC3) that generate the ATG12–ATG5 ligase activity responsible for the lipidation of LC3. Specifying the site of lipidation at the nascent membrane, the core proteins WIPI2 and ATG16L1 are pivotal in autophagosome elongation. Thus, WIPI2 binds first to membrane-bound PIP3 and then recruits the ATG12-ATG5-ATG16L1 complex which acts as an E3 ligase for the lipidation of LC3 [conjugation of phosphatidylethanolamine (PE)] that allow the growing of the phagophore. These two critical core proteins are targeted by E3 ligases mutated in neurological diseases. As discussed previously, Gigaxonin is a positive regulator of neuronal autophagy by fine-tuning ATG16L1 levels (Scrivo et al., 2019), while HUWE1 inhibits autophagy by degrading WIP2, a process under the direct control of mTORC1 (Wan et al., 2018). Before closure of the autophagosome, cytoplasmic material can be either engulfed in bulk autophagy, or selected through a process involving ubiquitination and recognition by autophagic receptors that contain both a LIR (LC3-interacting region) and an UBD (ubiquitin binding domain) motifs in selective autophagy. Mitophagy is the most extensively studied type of selective autophagy. Following the identification of the role of Parkin in the elimination of damaged mitochondria by autophagy (Narendra et al., 2008), concomitant publications revealed its mode of action: recruited by the kinase PINK1 onto altered organelles, Parkin gets activated and ubiquitinates several outer membrane components including mitofusin1/2, hence leading to the recognition of depolarized mitochondria by autophagy receptors (Gegg et al., 2010; Geisler et al., 2010; Poole et al., 2010; Ziviani et al., 2010). Interestingly, the FBXO7 E3 ligase, which is involved in Parkin-mediated mitophagy can promote mitophagy in similar way and compensate for parkin deficiency (Burchell et al., 2013). This general model may be enriched by other modulators, such as Malin and CHIP which interact with Parkin to enhance its stability and activity (Imai et al., 2002; Upadhyay et al., 2017), and NEDD4L which controls the ubiquitin-dependent degradation of Pink1 and negatively regulates autophagy (Huang et al., 2020), but their role in mitophagy remains to be demonstrated. Mitophagy can also be triggered independently of Parkin/Pink. In this context, HUWE1 is an inducer of AMBRA-mediated mitophagy, which does not require the main receptors as damaged organelles are addressed to autophagosomes by AMBRA, after activation of its LIR domain (Di Rita et al., 2018).

E3 ligases involved in neurological diseases control other types of selective autophagy. In xenophagy, Parkin and LRSAM1 mediate resistance to pathogens, by promoting ubiquitin coating of bacteria and their targeting to autophagosomes by receptors (Huett et al., 2012; Manzanillo et al., 2013). The HERC2 ligase was shown to negatively regulate ferritinophagy, the process that permits the release of iron through the breaking-down of ferritin by autophagy. Here, the action of HERC2 differs from the other types of selective autophagy, as the E3 ligase targets the ferritin receptor NCOA4 to ubiquitin-dependent degradation to prevent iron release (Mancias et al., 2015). Other studies show that E3 ligases can also act on receptors to potentiate selective autophagy. Indeed, HACE1 ubiquitinates the receptor OPTN, increasing its association with another receptor p62/SQSTM1 leading to enhanced autophagy, presumably by the creation of an autophagy receptor complex (Liu et al., 2014). Interaction of p62 with Malin (and Laforin) increases the activity of the E3 ligase, causing p62 ubiquitination (Sanchez-Martin et al., 2015).

Finally, future lines of investigations concern the last step of autophagy and upstream events of autophagy induction. Fusion of autophagosome to lysosome is powered by HOPS, a tethering complex which interacts with the autophagosomal syntaxin17. While all six subunits constituting the complex are required for fusion (Takats et al., 2014), VPS11 has been identified as an E3 ligase (Segala et al., 2019) but its mode of action and cooperation with other subunits remains to be determined. At the opposite side of the pathway, the pivotal kinase mTOR is a sensor of metabolic and nutrient stress regulating many cellular processes, including the repression of autophagy in normal conditions. Amongst the numerous E3 ligases that modulate mTOR signaling, two were investigated for autophagy readout: FBXW11 and TRIM32 maintain the repressive activity of mTOR on autophagy by degrading inhibitors of mTOR, respectively DEPTOR (Duan et al., 2011; Gao et al., 2011; Zhao et al., 2011) and RGS10 (Zhu et al., 2020). Directly under the control of mTOR, TFEB, a pivotal transcriptional regulator of autophagy genes is positively modulated by CHIP, which degrades preferentially its phosphorylated inactive forms and induces autophagy (Sha et al., 2017).

## Conclusion

E3 ubiquitin ligases are the genetic causes of a large number of neurological diseases. Mutations in these genes underlie a wide panel of neurological dysfunctions within the CNS and/or the PNS. While the study of the functions of mutated proteins is expanding, their E3 ligase nature brings substantial challenge for the deciphering of the complex pathological cascade at stake in disease. As the final enzyme delivering the ubiquitin moiety to specific targets, E3 ligases can tag hundreds of different substrates. Proteomic-driven or candidate approaches have been successfully used to unravel key functions but certainly, we have only a partial view of the global picture. Focusing on a rare recessive disease called giant axonal neuropathy (GAN), for its wide alteration of both the PNS and CNS, we discuss here 20 years of research on the genetic and functions of the Gigaxonin-E3 ligase. This led to an essential breakthrough in cell biology, where Gigaxonin is the 1st factor to date to control the degradation of the IF family, in and outside the nervous system. Exemplified by the massive aggregation of IF proteins throughout the body of patients, this pivotal cellular function is a signature for Gigaxonin across all experimental tools developed for GAN. The creation of mouse and, recently zebrafish models of the pathology led to major advance in the mechanisms underlying neuronal dysfunctions in disease. Thus, the recapitulation of the severe motor deficits of patients in zebrafish uncovered an unexpected role of Gigaxonin in controlling motor neuron birth and axonal outgrowth, through the regulation of Shh signaling. The *gan* zebrafish represents the first robust animal model for GAN, which enables the demonstration of the physiological relevance of Gigaxonin function in cell signaling and opens exciting perspective of a developmental origin in the settings of GAN. While the GAN knock-out mouse has a mild phenotype, the study of GAN neurons permitted to discover a pivotal role of Gigaxonin in autophagy, more specifically in the production of autophagosomes, through the control of steady-level of the ATG16L1 protein.

As discussed in this review, other E3 ligases causing neurological diseases act in similar pathways. Among those, autophagy is the most prevalent, with third of E3s controlling multiple steps of the autophagic machinery. While this depends largely on the focus given by research groups, this review highlights that 23% of the E3 ligases bear functions in multiples pathways, with numerous ones acting at multiple levels within the same pathway. In this context, Gigaxonin, TRIM32 and CHIP are crossing all categories, by targeting substrates in cytoskeleton, cell signaling and autophagy. This current view is undoubtedly going to expand in the future, but it already highlights the big challenge we are facing when thinking about therapy.

## Perspectives for Therapy

How can we integrate these various functions in therapeutic design, when working on reversing a single dysfunction is already a concern? Can one function be more biologically important than the others and be sufficient to alleviate neurological symptoms? Shall we reduce the complexity by targeting individual pathways or, on contrary embrace it by focusing on the E3 ligase, or on the physiological end point? To date, ongoing clinical trials for the neurological diseases discussed here concern symptomatic treatments for AS, or actions on the E3 ligases for AS and GAN. For AS, a phase I/II clinical trial strategy is based on silencing (by antisense oligonucleotide) a repressor transcript of the paternal *UBE3A* gene, to restore endogenous level of E6-AP [(Meng et al., 2015) and ClinicalTrials.gov Identifier: NCT04259281)], while GAN benefits from a phase I gene therapy approach (ClinicalTrials.gov Identifier: NCT02362438). Reintroducing Gigaxonin expression is in principle a promising approach, if appropriate level of the E3 ligase versus endogenous protein was determined. While we are assured that too much expression will generate serious deleterious effects, just mentioning the complete destruction of cytoskeleton, the risk of too little Gigaxonin is no impact on neurological signs. In absence of a robust GAN mouse and lack of demonstration that IF aggregation plays role in neurodegeneration, the benefit of this strategy (using a weak promotor) remains uncertain. To circumvent this, stabilizing the mutated E3 ligases (as it is the case for Gigaxonin) or modulating its activity (for dominant mode of inheritance) in disease constitutes an interesting approach, but knowledge on crystal structure are necessary for the latter. Another direction relies on the creation of powerful model systems to perform pharmacological screening, hence providing physiological relevance to candidate drugs. When it comes to mechanistic-driven approach, the pathologies discussed here may benefit from decades of pharmaceutical efforts done in other field, in particular cancer to potentiate or repress cell signaling, among which autophagy is certainly one prolific area of investigation (Dikic and Elazar, 2018). Thus, actual efforts in clinic favor strategies independent of mTOR, which exhibits deleterious side-effects due to its wide functions in other critical cellular processes, and search for more specificity, by targeting downstream regulators at every step of autophagy maturation, as well as selective autophagy (Boland et al., 2018).

As we are expanding our understanding of the functions of E3 ubiquitin ligases, there is a need to integrate them in a common ground in the future, to apprehend the complexity and the potential cross-talk between pathways in the settings of neurological symptoms. This constitutes a challenge, but the impact will be extremely high, by enriching our comprehension of cellular functions, and helping defining the best therapeutic strategies that can benefit multiples neurological diseases. Moreover, modulating activity of the wild-type E3 ligases is extremely relevant for many other diseases, if we consider their known roles in various functions such as DNA repair, cell division, apoptosis and immunity. Extensively studied in that respect, Itch represents a valuable therapeutic target for cancer, skin and immune diseases (Yin et al., 2020). Finally, methodologies exploiting the ubiquitination properties of E3 ligases, amongst which CRBN stands out propose now toolboxes (Schapira et al., 2019) allowing degrading, in principle any protein, including the undruggable.

## Author Contributions

PB wrote the manuscript, generated the table, and designed figures. LL executed complete drawing of figures and provided critical inputs into manuscript and figures. Both authors performed the bibliography search and reviewed the manuscript.

## Conflict of Interest

The authors declare that the research was conducted in the absence of any commercial or financial relationships that could be construed as a potential conflict of interest.
