# Supplementary material for: E3 Ubiquitin Ligases in Neurological Diseases: Focus on Gigaxonin and Autophagy
Source: Front Physiol. 2020 Oct 22;11:1022. doi: 10.3389/fphys.2020.01022 (PMC7642974; doi:10.3389/fphys.2020.01022)
Supplement: Supplementary file 1 [file Table_1.pdf]

	Mutations	Genetic	Country	Severity	kinky hair/ giant axons	References
Exon 1	V7Fs (ins)	ho (csg)	Tunisia	classical	yes/yes	(Bomont et al., 2000)
	V7Fs (del)	He[L]	Italy	mild	no/yes	(Bruno et al., 2004)
	R15S	ho (csg)	Tunisia	mild	no/yes	(Bomont et al., 2000)
	H33P	He[w]	China	mild	no/yes	(Wang et al., 2014)
	V35F	ho (csg)	Israël	mild	no/ni	(Aharoni et al., 2016)
	Q44X	He[t]	?	mild	no/ni	(Roth et al., 2013)
	A49E	ho	USA	classical	yes/yes	(Boizot et al., 2014)
	A49T	He[k]	Italy	classical	no/yes	(Bruno et al., 2004)
	A51P	ho (csg)	Pakistan	classical	yes/yes	(Houlden et al., 2007)
	S52G	He[d]	USA	classical	yes/yes	(Bomont et al., 2000)
	S52N	ho (csg)	Algeria	classical	yes/ni	(Echaniz-Laguna et al., 2020)
	P53L	He[w]	China	mild	no/yes	(Wang et al., 2014)
	Δex1-11	He[v]	?	?	yes/yes	(Mohammad et al., 2014)
Intron	IVS1-1 G>A	ho (csg)	Algeria	classical	yes/yes	(Koop et al., 2007)
Exon 2	Y71X	ho	Scotland	classical	no/yes	(Houlden et al., 2007)
	L75H	He[q]	China	classical	yes/yes	(Zhang and Zou, 2009)
	S79L	He[e]	Morocco	classical	yes/yes	(Bomont et al., 2000)
	«	He[ab]	China	classical	yes/yes	(Xu et al., 2020)
	V82F	He[a]	France	classical	yes/yes	(Bomont et al., 2000)
	I86F	He[g]	Germany	classical	yes/yes	(Bomont et al., 2003)
	Y89C	He[n]	Serbia	classical	yes/yes	(Koop et al., 2007)
	Δex2-9	ho (csg)	Algeria	classical	yes/ni	(Echaniz-Laguna et al., 2020)
	Δex2-11	He[r]	Belgium	classical	yes/yes	(Buysse et al., 2010)
Intron						
Exon 3	Δ114-119	He[g]	Germany	classical	yes/yes	(Bomont et al., 2003)
	F124C	ho (csg)	China	mild	no/yes	(Wang et al., 2014)
	R138H	ho	Algeria	mild	no/yes	(Bomont et al., 2000)
	R162X	ho (csg)	Japan	classical	yes/yes	(Akagi et al., 2012)
	«	ho (csg)	India	classical	yes/ni	(Garg et al., 2018)
	E169K	ho (csg)	Algeria	classical	no/yes	(Tazir et al., 2009)
	E180Fs	ho (csg)	India	classical	yes/ni	(Garg et al., 2018)
	V195F	He[p]	Germany	classical	yes/yes	(Koop et al., 2007)
	R201X	He[i]	Germany	classical	ni/ni	(Kuhlenbaumer et al., 2002)
Intron	IVS3+1G>T	He[p]	Germany	classical	yes/yes	(Koop et al., 2007)
Exon 4	R242X	ho (csg)	Turkey	classical	yes/yes	(Bomont et al., 2003)
	«	ho	India	classical	yes/yes	(Boizot et al., 2014)
	I244Fs	He[u]	?	classical	yes/ni	(Roth et al., 2013)
	R269Q	ho	Germany	classical	yes/yes	(Bomont et al., 2003)
	R269W	He[s]	China	mild	yes/yes	(Xu et al., 2013)
	«	He[u]	?	classical	yes/ni	(Roth et al., 2013)
	G270S	He[x]	Japan	mild	no/yes	(Koichihara et al., 2016)
Intron						
Exon 5	R293X	ho (csg)	Turkey	classical	yes/yes	(Demir et al., 2005)
	«	He[c]	Turkey	classical	yes/yes	(Bomont et al., 2000)
	Y299C	He[L]	Italy	mild	no/yes	(Bruno et al., 2004)
	L309R	ho (csg)	Tunisia	classical	yes/yes	(Bomont et al., 2000)
	P315L	He[k]	Italy	classical	no/yes	(Bruno et al., 2004)
	«	He[m]	England	classical	yes/yes	(Houlden et al., 2007)
	A324V	He[h]	New Zeland	mild	no/ni	(Boizot et al., 2014)
	E325K	ho (csg)	Israël	classical	yes/yes	(Abu-Rashid et al., 2013)
Intron						
Exon 6	G332R	ho (csg)	North Africa	classical	yes/yes	(Boizot et al., 2014)
	K338X	He[f]	France	classical	yes/ni	(Bomont et al., 2003)
	E362E[*]	ho (csg)	France	classical	yes/ni	(Echaniz-Laguna et al., 2020)
	Δex6-8	He[f]	France	classical	yes/yes	(Bomont et al., 2003)
Intron	IVS6+1G>C	He[j]	Italy	classical	yes/yes	(Bruno et al., 2004)

Exon 7	E392K	He[aa]	China	classical	yes/yes	(Cai et al., 2018)
	G368R	ho	Sri Lanka	classical	yes/yes	(Bomont et al., 2003)
	«	He[n]	Serbia	classical	yes/yes	(Koop et al., 2007)
	C393X	He[d]	USA	classical	yes/yes	(Bomont et al., 2000)
	W401X	He[b]	France	classical	yes/yes	(Bomont et al., 2000)
Intron	IVS7-1 G>A	ho	Japan	classical	yes/ni	(Miyatake et al., 2015)
8	I423T	He[i]	German	classical	ni/ni	(Kuhlenbaumer et al., 2002)
	W448L	ho (csg)	China	classical	no/yes	(Wang et al., 2014)
	R458W	ho (csg)	Algeria	classical	yes/ni	(Echaniz-Laguna et al., 2020)
Intron						
Exon 9	C464Y	He[h]	New Zeland	mild	no/ni	(Boizot et al., 2014)
	G474R	He[o]	Spain	classical	no/yes	(Koop et al., 2007)
	«	He[t]	?	mild	no/ni	(Roth et al., 2013)
	R477X	ho (csg)	Algeria	classical	yes/yes	(Bomont et al., 2003)
	«	ho (csg)	Algeria	classical	no/yes	(Bomont et al., 2003)
	«	He[o]	Spain	classical	no/yes	(Koop et al., 2007)
	«	ho (csg)	Algeria	classical	yes/yes	(Tazir et al., 2009)
	«	ho (csg)	Algeria	classical	yes/yes	(Tazir et al., 2009)
	«	ho (csg)	Algeria	classical	no/yes	(Tazir et al., 2009)
	«	ho (csg)	Algeria	classical	yes/yes	(Tazir et al., 2009)
	A461V	He[y]	Mexico	classical	yes/yes	(Normendez-Martinez et al., 2018)
	Q483X	ho (csg)	Tunisia	classical	yes/yes	(Bomont et al., 2000)
	E486K	ho (csg)	Tunisia	classical	yes/yes	(Bomont et al., 2000)
	«	He[b]	France	classical	yes/yes	(Bomont et al., 2000)
	«	He[r]	Belgium	classical	yes/yes	(Buysse et al., 2010)
	T489S	He[ab]	China	classical	yes/yes	(Xu et al., 2020)
	E493K	He[z]	India	classical	yes/ni	(Garg et al., 2018)
	«	ho (csg)	France	classical	yes/ni	(Echaniz-Laguna et al., 2020)
Intron	IVS9+1G>T	ho (csg)	Turkey	classical	yes/ni	(Demir et al., 2005)
	«	ho (csg)	Turkey	classical	yes/ni	(Demir et al., 2005)
	«	ho (csg)	Turkey	classical	yes/yes	(Incecik et al., 2015)
	«	ho (csg)	Turkey	classical	yes/yes	(Incecik et al., 2015)
	«	ho (csg)	Turkey	classical	ni/yes	(Edem et al., 2019)
10	W502X	ho (csg)	Pakistan	classical	yes/yes	(Houlden et al., 2007)
	L510X	He[j]	Italy	classical	yes/yes	(Bruno et al., 2004)
	F518Fs	He[m]	England	classical	yes/yes	(Houlden et al., 2007)
	Δex10-11	ho	USA	classical	yes/yes	(Boizot et al., 2014)
Intron						
Exon 11	R545C	ho	France	classical	yes/yes	(Bomont et al., 2000)
	R545H	ho	Finland	classical	yes/yes	(Koop et al., 2007)
	«	He[q]	China	classical	yes/yes	(Zhang and Zou, 2009)
	«	He[s]	China	mild	yes/yes	(Xu et al., 2013)
	«	He[aa]	China	classical	yes/yes	(Cai et al., 2018)
	R545L	He[y]	Mexico	classical	yes/yes	(Normendez-Martinez et al., 2018)
	T553Fs	ho (csg)	India	classical	yes/yes	(Nalini et al., 2008)
	P562A	ho	Italy	mild	yes/yes	(Bruno et al., 2004)
	R570Y	He[c]	Turkey	classical	yes/yes	(Bomont et al., 2000)
	A576E	He[x]	Japan	mild	no/yes	(Koichihara et al., 2016)
	«	He[z]	India	classical	yes/ni	(Garg et al., 2018)

Supplementary Table 1. Mutations identified in the *GAN* gene.
ho: homozygous mutation; csg: consanguineous family; He: heterozygous mutation (compound mutations for the same patient are indicated by letter in [exponent]); E362E*: silent mutation (at the last amino acid of exon 6) affects splicing and leads to premature stop codon; ni: non investigated. PS: we apologize to authors but 3 publications were excluded from the table for the following reasons: Leung *et al.,* BMC genet 2007 (absence of clinical data, no screen in controls); Wang *et al.*, Zhonghua Yi Xue Yi Chuan Xue Za Zhi 2016 (article in chinese); Almeida *et al.,* An Bras Dermatol 2016 (incomplete data). Also, in Koop et al., Neuromscul Disord 2007: variant found in the promotor in family 9 was not included (uncertain & other allele not identified).

References

Abu-Rashid, M., Mahajnah, M., Jaber, L., Kornreich, L., Bar-On, E., Basel-Vanagaite, L., Soffer, D., Koenig, M., and Straussberg, R. (2013). A novel mutation in the GAN gene causes an intermediate form of giant axonal neuropathy in an Arab-Israeli family. European journal of paediatric neurology : EJPN : official journal of the European Paediatric Neurology Society *17*, 259-264.

Aharoni, S., Barwick, K.E., Straussberg, R., Harlalka, G.V., Nevo, Y., Chioza, B.A., McEntagart, M.M., Mimouni-Bloch, A., Weedon, M., and Crosby, A.H. (2016). Novel homozygous missense mutation in GAN associated with Charcot-Marie-Tooth disease type 2 in a large consanguineous family from Israel. BMC medical genetics *17*, 82.

Akagi, M., Mohri, I., Iwatani, Y., Kagitani-Shimono, K., Okinaga, T., Sakai, N., Ozono, K., and Taniike, M. (2012). Clinicogenetical features of a Japanese patient with giant axonal neuropathy. Brain Dev *34*, 156-162.

Boizot, A., Talmat-Amar, Y., Morrogh, D., Kuntz, N.L., Halbert, C., Chabrol, B., Houlden, H., Stojkovic, T., Schulman, B.A., Rautenstrauss, B.*, et al.* (2014). The instability of the BTB-KELCH protein Gigaxonin causes Giant Axonal Neuropathy and constitutes a new penetrant and specific diagnostic test. Acta neuropathologica communications *2*, 47.

Bomont, P., Cavalier, L., Blondeau, F., Ben Hamida, C., Belal, S., Tazir, M., Demir, E., Topaloglu, H., Korinthenberg, R., Tuysuz, B.*, et al.* (2000). The gene encoding gigaxonin, a new member of the cytoskeletal BTB/kelch repeat family, is mutated in giant axonal neuropathy. Nat Genet *26*, 370-374.

Bomont, P., Ioos, C., Yalcinkaya, C., Korinthenberg, R., Vallat, J.M., Assami, S., Munnich, A., Chabrol, B., Kurlemann, G., Tazir, M.*, et al.* (2003). Identification of seven novel mutations in the GAN gene. Hum Mutat *21*, 446.

Bruno, C., Bertini, E., Federico, A., Tonoli, E., Lispi, M.L., Cassandrini, D., Pedemonte, M., Santorelli, F.M., Filocamo, M., Dotti, M.T.*, et al.* (2004). Clinical and molecular findings in patients with giant axonal neuropathy (GAN). Neurology *62*, 13-16.

Buysse, K., Vergult, S., Mussche, S., Ceuterick-de Groote, C., Speleman, F., Menten, B., Lissens, W., and Van Coster, R. (2010). Giant axonal neuropathy caused by compound heterozygosity for a maternally inherited microdeletion and a paternal mutation within the GAN gene. Am J Med Genet A *152A*, 2802-2804.

Cai, S., Lin, J., Liu, Y.Q., Lu, J.H., and Zhao, C.B. (2018). Giant Axonal Neuropathy with Unusual Neuroimagings Caused by Compound Heterozygous Mutations in GAN Gene. Chinese medical journal *131*, 2371-2372.

Demir, E., Bomont, P., Erdem, S., Cavalier, L., Demirci, M., Kose, G., Muftuoglu, S., Cakar, A.N., Tan, E., Aysun, S.*, et al.* (2005). Giant axonal neuropathy: clinical and genetic study in six cases. J Neurol Neurosurg Psychiatry *76*, 825-832.

Echaniz-Laguna, A., Cuisset, J.M., Guyant-Marechal, L., Aubourg, P., Kremer, L., Baaloul, N., Verloes, A., Beladgham, K., Perrot, J., Francou, B.*, et al.* (2020). Giant axonal neuropathy: a multicenter retrospective study with genotypic spectrum expansion. Neurogenetics *21*, 29-37.

Edem, P., Karakaya, M., Wirth, B., Okur, T.D., and Yis, U. (2019). Giant axonal neuropathy: A differential diagnosis of consideration. The Turkish journal of pediatrics *61*, 275-278.

Garg, M., Kulkarni, S.D., Hegde, A.U., Desai, M., and Sayed, R.J. (2018). Giant Axonal Neuropathy: Clinical, Radiological, and Genetic Features. Annals of Indian Academy of Neurology *21*, 304-308.

Houlden, H., Groves, M., Miedzybrodzka, Z., Roper, H., Willis, T., Winer, J., Cole, G., and Reilly, M.M. (2007). New mutations, genotype phenotype studies and manifesting carriers in giant axonal neuropathy. J Neurol Neurosurg Psychiatry *78*, 1267-1270.

Incecik, F., Herguner, O.M., Ceylaner, S., Zorludemir, S., and Altunbasak, S. (2015). Giant axonal disease: Report of eight cases. Brain Dev *37*, 803-807.

Koichihara, R., Saito, T., Ishiyama, A., Komaki, H., Yuasa, S., Saito, Y., Nakagawa, E., Sugai, K., Shiihara, T., Shioya, A.*, et al.* (2016). A mild case of giant axonal neuropathy without central nervous system manifestation. Brain Dev *38*, 350-353.

Koop, O., Schirmacher, A., Nelis, E., Timmerman, V., De Jonghe, P., Ringelstein, B., Rasic, V.M., Evrard, P., Gartner, J., Claeys, K.G.*, et al.* (2007). Genotype-phenotype analysis in patients with giant axonal neuropathy (GAN). Neuromuscul Disord *17*, 624-630.

Kuhlenbaumer, G., Young, P., Oberwittler, C., Hunermund, G., Schirmacher, A., Domschke, K., Ringelstein, B., and Stogbauer, F. (2002). Giant axonal neuropathy (GAN): case report and two novel mutations in the gigaxonin gene. Neurology *58*, 1273-1276.

Miyatake, S., Tada, H., Moriya, S., Takanashi, J., Hirano, Y., Hayashi, M., Oya, Y., Nakashima, M., Tsurusaki, Y., Miyake, N.*, et al.* (2015). Atypical giant axonal neuropathy arising from a homozygous mutation by uniparental isodisomy. Clinical genetics *87*, 395-397.

Mohammad, S.S., Lau, C., Burke, C., McCallum, N., and Robertson, T. (2014). Giant axonal neuropathy diagnosed on skin biopsy. Journal of clinical

neuroscience : official journal of the Neurosurgical Society of Australasia *21*, 865-867.

Nalini A, G.N., Yasha TC, Ravishankar S, Urtizberea A, Huehne K, Rautenstrauss B (2008). Clinical, pathological and molecular findings in two siblings with giant axonal neuropathy (GAN): report from India. Eur J Med Genet. *51*, 426-435.

Normendez-Martinez, M.I., Monterde-Cruz, L., Martinez, R., Marquez-Harper, M., Esquitin-Garduno, N., Valdes-Flores, M., Casas-Avila, L., de Leon-Suarez, V.P., Romero-Diaz, V.J., and Hidalgo-Bravo, A. (2018). Two novel mutations in the GAN gene causing giant axonal neuropathy. World journal of pediatrics : WJP *14*, 298-304.

Roth, L.A., Johnson-Kerner, B.L., Marra, J.D., Lamarca, N.H., and Sproule, D.M. (2013). The absence of curly hair is associated with a milder phenotype in Giant Axonal Neuropathy. Neuromuscul Disord.

Tazir, M., Nouioua, S., Magy, L., Huehne, K., Assami, S., Urtizberea, A., Grid, D., Hamadouche, T., Rautenstrauss, B., and Vallat, J.M. (2009). Phenotypic variability in giant axonal neuropathy. Neuromuscul Disord *19*, 270-274.

Wang, L., Zhao, D., Wang, Z., Zhang, W., Lv, H., Liu, X., Meng, L., and Yuan, Y. (2014). Heterogeneity of axonal pathology in Chinese patients with giant axonal neuropathy. Muscle Nerve *50*, 200-205.

Xu, M., Da, Y.W., Liu, L., Wang, F., and Jia, J.P. (2013). Giant axonal neuropathy caused by a novel compound heterozygous mutation in the gigaxonin gene. J Child Neurol *28*, 1316-1319.

Xu, X., Yang, X., Su, Z., Wang, H., Li, X., Sun, C., Wang, W., Chen, Y., Zhang, C., Zhang, H., *et al.* (2020). Identification of Novel Compound Heterozygous Mutations in the GAN Gene of a Chinese Patient Diagnosed With Giant Axonal Neuropathy. Frontiers in neuroscience *14*, 85.

Zhang, L.P., and Zou, L.P. (2009). Clinical and genetic studies in a Chinese family with giant axonal neuropathy. J Child Neurol *24*, 1552-1556.
